# Polyphenols and Their Metabolites in Renal Diseases: An Overview

**DOI:** 10.3390/foods11071060

**Published:** 2022-04-06

**Authors:** Íris Guerreiro, Cíntia Ferreira-Pêgo, Diogo Carregosa, Cláudia N. Santos, Regina Menezes, Ana S. Fernandes, João G. Costa

**Affiliations:** 1 CBIOS—Universidade Lusófona’s Research Center for Biosciences & Health Technologies, Campo Grande 376, 1749-024 Lisboa, Portugal; p5863@ulusofona.pt (Í.G.); cintia.pego@ulusofona.pt (C.F.-P.); regina.menezes@ulusofona.pt (R.M.); ana.fernandes@ulusofona.pt (A.S.F.); 2 CEDOC, Chronic Diseases Research Center, NOVA Medical School, Faculdade de Ciências Médicas, Universidade NOVA de Lisboa, Campo dos Mártires da Pátria, 130, 1169-056 Lisboa, Portugal; diogo.carregosa@nms.unl.pt (D.C.); claudia.nunes.santos@nms.unl.pt (C.N.S.); 3 iBET, Instituto de Biologia Experimental e Tecnológica, Apartado 12, 2781-901 Oeiras, Portugal

**Keywords:** polyphenols, metabolites, renal diseases, acute kidney injury, chronic kidney disease, diabetic nephropathy, renal cancer, drug-induced nephrotoxicity

## Abstract

Kidney diseases constitute a worldwide public health problem, contributing to morbidity and mortality. The present study aimed to provide an overview of the published data regarding the potential beneficial effects of polyphenols on major kidney diseases, namely acute kidney injury, chronic kidney disease, diabetic nephropathy, renal cancer, and drug-induced nephrotoxicity. This study consists of a bibliographical review including in vitro and in vivo studies dealing with the effects of individual compounds. An analysis of the polyphenol metabolome in human urine was also conducted to estimate those compounds that are most likely to be responsible for the kidney protective effects of polyphenols. The biological effects of polyphenols can be highly attributed to the modulation of specific signaling cascades including those involved in oxidative stress responses, anti-inflammation processes, and apoptosis. There is increasing evidence that polyphenols afford great potential in renal disease protection. However, this evidence (especially when in vitro studies are involved) should be considered with caution before its clinical translation, particularly due to the unfavorable pharmacokinetics and extensive metabolization that polyphenols undergo in the human body. Future research should consider polyphenols and their metabolites that indeed reach kidney tissues.

## 1. Introduction

Kidney diseases constitute a worldwide public health problem, contributing to morbidity and mortality from non-communicable diseases, both as a direct cause and as a risk factor for cardiovascular disease [1]. The burden of renal pathologies is rising year after year in different regions of the world [2]. This increase is associated with higher mortality and treatment costs [2], demanding great attention in terms of global health policies. The variations in incidence, prevalence, and outcomes of renal diseases may depend on several biological, socioeconomic, and behavioral risk factors [3]. Several studies have explored the role of diet in the context of chronic kidney diseases or renal cancer [4,5]. However, the effects of dietary interventions on the outcome of kidney diseases remain to be clarified. Polyphenols are among the dietary compounds studied in this context. Polyphenols are characterized by the presence of one or more hydroxyl groups attached to aromatic rings. Other compounds are commonly integrated into this group, such as phenolic acids or stilbenes. For this reason, the denomination “(poly)phenols” is sometimes used in the literature. However, we will adopt the word “polyphenols” as an umbrella term throughout the manuscript for simplicity.

Despite the lack of conclusive epidemiological data, numerous in vitro and in vivo studies have dealt with the effects of polyphenols and kidney diseases. In addition to a dietary approach, polyphenols have been explored as potential therapeutic/nutraceutical agents against kidney diseases [6,7]. In this context, some studies use high doses, different administration routes and specific drug delivery systems to target kidney tissues [8,9]. The interest in these compounds is supported essentially by their ability to modulate redox and inflammatory pathways. Scientific evidence suggests that reactive oxygen species (ROS) and inflammation play a key role in the pathophysiologic processes of renal diseases. The kidney is an organ particularly vulnerable to ROS attack [10] and oxidative damage is associated with a wide range of renal impairments, including acute renal failure [11], obstructive nephropathy [12], glomerular damage [13] or chronic renal failure [14]. Therefore, dietary and pharmacological antioxidant/anti-inflammatory interventions could attenuate renal damage [15]. It should be noted that the use of the term antioxidant throughout the manuscript refers to its broader definition, instead of the classical view of antioxidants as merely scavenging/reducing agents. In biology and medicine, antioxidants can be defined as any substance that can prevent, reduce, or repair the ROS-induced damage of a target biomolecule, including via indirect mechanisms such as the upregulation of nuclear factor E2-related factor 2 (Nrf2) [16], which appears to be highly relevant in polyphenols mode of action.

The aim of this study was to provide an overview of the published data regarding the potential beneficial effects of polyphenols on major kidney diseases not distinguishing the nutritional and pharmacological perspectives of those studies. For that, a literature search in English was conducted in Medline, Scopus, and Web of Science, to identify and select relevant studies evaluating the effects of pure polyphenols on renal diseases, with a special emphasis on those published in the last two decades. The search terms included: “polyphenol(s)”, “flavonoid” or “anthocyanin”, and “acute kidney injury”, “diabetic nephropathy”, “renal cancer” or “drug-induced nephrotoxicity”. We do not attempt to present an exhaustive and detailed review of all studies available or to compare experimental details (e.g., concentrations, doses, or methodologies). On the contrary, we intend to give an overview emphasizing the importance of considering the polyphenols fate in the human body when designing a study. This review includes in vitro and in vivo studies dealing with the effects of individual compounds, rather than mixtures, such as extracts or food stuff (Section 2.1, Section 2.2, Section 2.3, Section 2.4, Section 2.5). An analysis of the polyphenol metabolome in human urine was also conducted, in order to estimate those compounds that are most likely to be responsible for the kidney protective effects of polyphenols (Section 3).

## 2. Implication of Polyphenols in Renal Pathophysiology

### 2.1. Acute Kidney Injury

Acute kidney injury (AKI) is characterized by a loss of kidney function with an increase in serum creatinine, decrease in urinary output, or both for a period until 7 days [17,18]. AKI occurs in approximately 10–15% of patients admitted to the hospital, and its incidence in intensive care can even exceed 50% [17]. AKI is not a single disease entity, but a part of a heterogeneous functional group of disorders that can occur in the setting of acute or chronic illness [18,19]. Nevertheless, despite its complexity, AKI is usually seen as a single disease and classified according to anatomical categories [17]. In AKI, kidney homeostasis is disrupted and in severe cases, it can lead to multiorgan failure being potentially lethal [18]. AKI may be induced by cisplatin, an anticancer drug, and cisplatin treatment is a well-established model to study this kidney injury. The role of polyphenols in this particular cisplatin-induced AKI condition will be discussed in the section about drug-induced nephrotoxicity (2.5).

Many studies have shown that polyphenols can act against various factors that are linked to AKI. Resveratrol (3,5,4′-trihydroxystilbene) is a natural polyphenol that belongs to the stilbenes class. It is present in many plants, and it is the most studied polyphenol that has shown potential protection against AKI. Resveratrol ameliorated several kidney function markers and pathological damage of AKI. Resveratrol showed its effectiveness against AKI through the reduction of ROS in HK-2 human renal cells [20]. Additionally, resveratrol reduced inflammatory (e.g., TNF-α and IL-1β) and kidney injury (e.g., KIM-1) markers, and reversed the alterations of apoptosis-associated proteins (e.g., Bcl-2 and Bax) in different in vitro and in vivo models [21,22,23]. Sepsis is the most common cause of severe AKI in individuals that are extremely ill [17]. In this sense, some studies with septic AKI animal models have also been used to study the beneficial effects of polyphenols in this pathophysiological condition. Resveratrol decreased the mortality rate of septic rodents, alleviated AKI, improved renal microcirculation, protected the tubular epithelium, ameliorated oxidative stress and mitochondrial function, and reduced the inflammatory response [24,25,26]. Mitochondrial dysfunction is one of the characteristics of AKI. The resveratrol glycoside (resveratrol-3-*O*-β-mono-d-glucoside), also known as polydatin or piceid, protected renal tubular epithelial cell mitochondria from dysfunction, reduced inflammatory and oxidative stress parameters, and prolonged survival in a rat model of sepsis-induced AKI [27]. Gallic acid, a phenolic acid present in a large number of plants, also showed significant protection against renal ischemia/reperfusion (I/R)-induced AKI in a rat model [28]. Some studies revealed the antioxidant properties and the improvement in renal function by epigallocatechin-3-gallate (EGCG), the major flavanol present in the tea plant *Camellia sinensis*. EGCG reduced ROS and renal damage by iron overload, acting as an iron chelator, reducing hypoxic damage and oxidative and nitrosative stress [29,30]. EGCG also ameliorated cardiopulmonary bypass-induced AKI in diabetic rats, prevented renal tubular damage, and reduced the level of kidney injury and oxidative stress biomarkers [31]. Another study showed that EGCG could protect the kidney from I/R injury, reducing macrophage infiltration, renal fibrosis, and several molecules involved with an inflammatory response [32]. Curcumin, a biologically active polyphenolic compound obtained from the rhizomes of the plant *Curcuma longa* that is present in several spices, significantly decreased the rate of apoptosis and protected renal cells against I/R-induced AKI [33]. Ellagic acid, a natural polyphenol compound present in food (e.g., chestnut, pomegranate, and blackberry), attenuated the renal ischemia/reperfusion (I/R) injury, a primary reason for AKI, and preserved renal cell function in rats. Additionally, ellagic acid suppressed the levels of inflammatory, oxidative stress, and apoptosis markers in an I/R rat model [34]. Honokiol, a natural polyphenol from the traditional Chinese herb *Magnolia officinalis* attenuated sepsis-associated AKI and ameliorated oxidative stress and inflammatory signals in NRK-52E cells, as well as in a rat model with cecal ligation and puncture (CLP)-induced oxidative stress and inflammatory cytokine production [35].

### 2.2. Chronic Kidney Disease

Chronic kidney disease (CKD) is a condition defined as persistent alterations in kidney structure, function, or both of at least 3 months duration [36,37]. CKD is associated with urine and structural abnormalities and impaired excretory renal function, which are suggestive of irreversible loss of functional nephrons [37]. CKD arises from many heterogeneous disease pathways, with diabetes and hypertension being the main causes [36,37]. The prevalence of CKD varies between 7–12% worldwide [37]. The most relevant pathophysiologic changes include glomerular sclerosis, tubular atrophy, and interstitial fibrosis [36]. It is common to use animal models in which CKD is associated with diabetes. Nonetheless, the role of polyphenols in models of this particular kidney condition will be presented in the next Section 2.3. 

The beneficial impact of polyphenols in CKD has been explored, mainly due to their antioxidant and anti-inflammatory properties. In a recent study, resveratrol alleviated the increase in markers of kidney function, the presence of glomerular sclerosis, and the tubulointerstitial fibrosis induced in nephrectomy rodent models [38]. In another study using a mice model, resveratrol treatment inhibited oxidative stress and renal interstitial fibrosis [39]. Mitochondrial dysfunction is one of the cellular alterations of CKD. In a study performed by Hui et al., (2017), resveratrol attenuated glomerular injury in the remnant kidney of nephrectomized rats and also improved mitochondrial function in vitro and in vivo [40]. Skeletal muscle atrophy is one of the clinical characteristics of CKD. Resveratrol prevented the increase in expression of important pathophysiologic proteins (e.g., MuRF1) in vitro and attenuated muscle atrophy induced by CKD in a rodent model [41]. EGCG exhibited renoprotective effects in mice with unilateral ureteral obstruction, by reducing the inflammatory response and oxidative stress [42]. Moreover, the preventive role of EGCG in CKD and renal fibrosis has also been discussed by its ability to preserve mitochondrial function, antiapoptotic effects, and anti-epithelial mesenchymal transition properties [43]. I/R-induced AKI can lead to renal fibrosis, which is a relevant risk factor for CKD. In a study performed by Hongtao et al. [44], curcumin alleviated I/R-induced late kidney fibrosis in a mouse model [44]. Salvianolic acid A demonstrated antioxidant effects in vitro and reduced kidney injury, inflammation, and oxidative stress markers in a nephrectomized rat model [45].

### 2.3. Diabetic Nephropathy

Diabetes is a highly prevalent chronic disease affecting more than four hundred million adults worldwide. The disease compromises several body functions, including diabetic nephropathy (DN), also referred as diabetic kidney disease (DKD). DN is among the most common causes of morbidity and mortality in individuals with diabetes as well as the main culprit for end-stage renal disease in the world. With multifactorial and complex pathophysiology, DN management has been considered a major challenge for physicians and the pharmaceutical industry. At the cellular level, DN is associated with several cellular pathways including autophagy dysregulation, oxidative stress, hypoxia, inflammation, and overactive renin-angiotensin-aldosterone system (RAAS) [46,47]. The multitarget effects and broad spectrum of health benefits of polyphenols have pointed these compounds as promising therapeutic intervenients to fight the multiple complications of such complex diseases. In fact, in vitro and preclinical studies have shown that stilbenes, flavonoids (in particular, anthocyanins), and lignans slow the progression of kidney damage and prevent ischemic events and DN [48]. 

The stilbene resveratrol has gained a great deal of attention thanks to its multiple, yet controversial, actions as an antioxidant, anti-inflammatory, anti-diabetic molecule particularly towards the dysfunction of the renal system in diabetes [49,50,51]. The nephroprotective action of resveratrol as determined in animal and in vitro studies includes the modulation of oxidative stress [50], advanced glycation end-product (AGE) cytotoxicity [52], autophagy, endoplasmic reticulum (ER) stress, apoptosis [53,54,55], lipotoxicity, mitochondrial dysfunction, angiogenesis [50], and inflammation [56]. Remarkably, resveratrol inhibited lipopolysaccharide (LPS)-induced rat glomerular mesangial cells proliferation and inflammation, suggesting that it may prevent and/or delay mesangial cell fibrosis independently of its hypoglycemic activity [57]. Additionally, interestingly, resveratrol and ramipril co-treatment showed reversibility of glomerulosclerosis in early stage DN, supporting the efficiency of a combined therapeutic strategy in the early DN intervention [58]. Polydatin has been also shown to protect against renal dysfunction in DN by mechanisms including the attenuation of mitochondrial, reversion of apoptosis, suppression of oxidative stress, and mitigation of renal inflammation and fibrosis [59,60,61,62,63,64]. Punicalagin, the major hydrolysable tannin from pomegranate, whose metabolism involves the formation of gallic acid, has been also associated with DN protection. The attenuation of inflammation and pyroptosis was pointed to as the molecular mechanisms underlying punicalagin-mediated effects [65]. Cyanidin 3-glucoside is the most widespread flavonoid from the anthocyanin sub-class. Its protective effects against DN have been associated with the alleviation of apoptosis, oxidative stress [66,67,68,69], improvement of autophagy, inhibition of epithelial-mesenchymal transition (EMT) [69], and attenuation of inflammation [66,70]. Protocatechuic acid, also referred as 3,4-dihydroxybenzoic acid, is a phenolic acid from the hydroxybenzoic acids sub-class and a major polyphenol metabolite derived from anthocyanins metabolism. Its reported beneficial effects against DN include the inhibition of high glucose (HG)-induced human mesangial cells proliferation and oxidative stress [71]. As stilbenes and anthocyanins, formononetin, a flavonoid from the isoflavonoids sub-class, was shown to alleviate oxidative stress burden in the kidney of diabetic animals, which may contribute to the control of hyperglycemia and insulin resistance and the reduction of triglyceride, cholesterol, creatinine, and urea in the blood [72]. The flavanol quercetin has also been associated with several protective activities against DN. It was shown to antagonize glucose fluctuation-induced renal injury by suppressing aerobic glycolysis [73], to inhibit proliferation in HG–treated glomerular mesangial cells and in early DN mouse [74], and to prevent the initiation and progression of DN in diabetic mice by improving the renal accumulation of lipid bodies [75]. Interestingly, quercetin liposomes improved DN biochemistry and pathological changes in a higher extent than non-encapsulated quercetin, which was attributed to the maintenance of quercetin in higher concentrations in the plasma [76]. Another study comparing the nephroprotective activities of quercetin and quercetin/nanoparticle complex revealed that both treatments prevented kidney pathological damage and improved renal function, alleviated renal oxidative stress, and attenuated inflammatory processes with a greater effect in animals treated with quercetin/nanoparticle complex [77], further supporting the efficacy of vehiculation strategies to improve the phenolics bioactivity towards DN. Quercetin 3-*O*-galactoside, also known as hyperoside or hyperin, exhibits bioactive properties related to the improvement of cell injury and relieve the signs of renal dysfunction via targeting the miR-499-5p/APC axis [78]. Additionally, dihydroquercetin was shown to mitigate the renal histopathological lesions associated with DN by mechanisms that may involve oxidative stress and inflammation suppression [79]. The nephroprotective action of the glycosyloxyflavone myricitrin, another compound belonging to flavonols, was found to be associated with the mitigation of oxidative stress as investigated both in vitro and in vivo, as well as to prevent renal inflammation [80,81]. Remarkably, vehiculation of myricitrin using solid lipid nanoparticles was shown to increment myricitrin effects in vivo [81]. EGCG has been associated with the modulation of several renoprotective signaling pathways [82]. It has shown beneficial effects towards DN via modulating oxidative stress responses [83,84,85]. An in vivo study investigating the role of EGCG and methylated EGCG, a metabolite with greater bioavailability than EGCG, on diacylglycerol kinase α (DGKα)-mediated alleviation of DN unveiled that both catechins ameliorated albuminuria and attenuated HG-induced podocytes loss by preventing a decrease in focal adhesion [86]. Moreover, it was observed that EGCG alleviates renal fibrosis, a histopathological feature of DN [87]. In addition, it was shown that ECGC promoted HG-podocyte cell proliferation, decreased apoptosis, and attenuated the expression of ER stress markers, suggesting that EGCG may protect podocytes against apoptosis via suppressing ER stress [88]. Testing of epicatechin and the metabolites derived from flavonoid intake, 2,3-dihydroxybenzoic acid, 3′,4′-dihydroxyphenylacetic acid and 3-(3′-hydroxyphenyl)propanoic acid, towards the prevention of inflammation and the accompanying redox imbalance in HG- and lipopolysaccharide-induced renal proximal tubular cells revealed that NOX-4/p38 plays a crucial role on the protective effect of epicatechin and 2,3-dihydroxybenzoic acid [89]. Procyanidin B2 is flavan-3-ol dimer composed of two molecules of (−)-epicatechin. Its reported protective effects on DN have been associated with the relief of HG-podocyte injury in vivo [90], apoptosis, mitochondrial dysfunction [91] and inflammation. It was also shown to reverse HG-induced EMT-associated morphological changes in renal tubular epithelial cells. At last, (+)-catechin was shown to ameliorate renal dysfunction in vivo through the inhibition of AGEs formation and inflammatory pathways via methylglyoxal trapping [92]. Oligonol, a phenolic product derived from lychee fruit, is produced by a manufacturing process that converts polyphenol polymers into oligomers being therefore rich in catechin-type monomers and oligomers of proanthocyanidins. It was shown to attenuate inflammation and glomerular hypertrophy in vivo and to suppress renal oxidative stress [93]. Its pleiotropic action was also associated with protection against AGE formation and apoptosis [94]. A plethora of oligonol renoprotective activities has been discussed elsewhere [95]. The effects of bergenin, a C-glycoside of 4-*O*-methylgallic acid also known as cuscutin, against DN include the downregulated oxidative stress thereby inhibiting extracellular matrix generation in glomerular mesangial cells and contributing to the alleviation of nephropathy both in vivo and in vitro [96]. Sinapic acid, a polyphenol metabolite also present in foodstuffs, was shown to be nephroprotective via regulation of oxidative stress and inflammation. The nuclear factor erythroid 2-related factor 2/heme oxygenase 1 (NRF2/HO-1) pathway appears as the main target underlying sinapic acid bioactivity [97]. Oleuropein, belonging to the polyphenols sub-class of tyrosols, is the most common phenolic compound in olives. Reduction of body weight, alleviation of kidney injury, and decrease of inflammatory response after oleuropein treatment was associated with the inhibition of cell apoptosis in renal sections and alleviation of kidney oxidative stress [98]. Regarding other polyphenols that do not belong to the classes referred before, salvianolic acid A renoprotective activities, namely the restoration of glomerular endothelial function and alleviation of renal structural deterioration, were shown to be associated with the suppression AGEs-induced rearrangement of actin cytoskeleton, attenuation of AGEs-induced oxidative stress with consequent alleviation of inflammation and restoration of autophagy, as determined in glomerular endothelial cells and diabetic rats [99]. In vivo, treatment with the natural biphenolic compound, honokiol, mitigates ROS production which translates into the attenuation of renal dysfunction markers such as albuminuria, glomerular damage, and podocyte injury [100]. 

### 2.4. Renal Cancer

Kidney cancers are the group of malignancies representing the 15th most common type of cancer worldwide, responsible for 2.2% of all new cases of cancer and nearly 180,000 deaths, in 2020 [101]. Renal cell carcinoma (RCC) is the most common type, comprising nearly 90% of all kidney cancers and representing a panel of heterogeneous tumor subtypes. The classification recognized by the World Health Organization (WHO) depicts histopathological dissimilarities between these tumors, establishing sixteen different subtypes of RCC. Clear cell renal cell carcinoma (ccRCC) is the most expressive of the RCC subtypes, generally initiating at the epithelial cells of the proximal tubule, as a result of the manifestation of different genetic events [102,103,104].

Evidence has supported the potential anticancer effects of polyphenols on different types of cancer, including RCC [70,105,106]. Resveratrol is among the most attractive polyphenols regarding cancer protection, and it has been suggested as a promising anti-cancer agent on RCC. Treatment of human renal cancer cells 786-O with resveratrol inhibited cell proliferation in a concentration-dependent manner and suppressed the expression of the vascular endothelial growth factor (VEGF) gene [107]. In 2015, Chen and colleagues reported that resveratrol was able to control tumor growth and modulate the tumor microenvironment in a mouse renal tumor model [108]. Resveratrol was further shown to inhibit cell proliferation, induce cell cycle arrest on S phase, suppress invasive phenotype and colony formation activity on RCC cell models, by preventing the activation of Janus activated kinases (JAKs) 1 and 2, and Src kinases, therefore blocking the JAK/STATs (Janus kinase/signal transducer and activator of transcription) signaling [109]. Other pathways involved in tumor progression have been identified as targets of resveratrol in RCC cell models. AT1R/VEGF pathway (Angiotensin II type 1 receptor/Vascular endothelial growth factor) was impaired in the presence of resveratrol, through downregulation of Angiotensin II, AT1R (Angiotensin II type 1 receptor), VEGF and ciclooxigenase-2 expression [110]. Moreover, this polyphenol was able to modulate the inflammatory response by inhibiting the activity of NOD-, LRR- and pyrin domain-containing protein 3 (NLRP3) inflammasome, which is highly expressed on RCC [106,111]. Akt (protein kinase B), ERK1/2 (Extracellular signal-regulated kinase 1/2) and p53/AMPK/mTOR (cellular tumor antigen p53/AMP-activated Protein Kinase/mammalian target of rapamycin)-induced autophagy signaling pathways were also reported as targets of resveratrol on RCC, leading to the suppression of cell proliferation, migration, invasion, and induction of apoptosis in a concentration and time-dependent manner [112,113]. This compound has also been shown to affect epigenetic mechanisms such as in impairment of histone acetylation leading to decreased activation of MMP-2/-9 (Matrix metalloproteinases 2 and 9) [114]. Resveratrol effects on RCC cell proliferation and apoptosis were shown to be enhanced in the presence of autophagy inhibitors [115]. In a different perspective, the combination of chemotherapeutic agents with resveratrol was also shown to have beneficial effects. Resveratrol enhances the apoptotic effect of sorafenib in 786-O cells, through blockage of the Jak2/STATs pathway [109]. In paclitaxel-resistant RCC cells, resveratrol increased sensitivity to this chemotherapeutic drug, through inhibition of the PI3K/AKT (Phosphatidylinositol 3-kinase/Protein kinase B) pathway [116]. Another approach has explored the potential anticancer effects of resveratrol combined with sitagliptin. A synergistic effect leading to the impairment of STAT3/NFKβ (signal transducer and activator of transcription/nuclear factor kappa-light-chain-enhancer of activated B cells) and NFR2/HO-1 pathways and promotion of apoptosis was found [117]. Zeng et al. have demonstrated that resveratrol plus a fiber-modified replication-deficient adenovirus Ad5/35-TRAIL (tumor necrosis factor-related apoptosis-inducing ligand) significantly inhibited RCC xenograft growth in nude mice [118]. Interestingly, it has been suggested that resveratrol may regulate the expression of tumor suppressor genes by interaction with miRNAs, including mir-21, an important player in renal tumor development [119]. Another polyphenol, EGCG, has been reported to promote the expression of different tumor suppressor genes by interacting with miR-210, which is downregulated in several types of tumors, including RCC [120]. EGCG has been reported to inhibit cell proliferation and induce apoptosis in 786-O cells, by inducing the overexpression of TFPI-2 (tissue factor pathway inhibitor-2) [121]. Chen et al. observed an EGCG-derived decrease in migration and invasion abilities, associated with the downregulation of MMP-2/-9 [122]. EGCG was also found to increase the sensitivity of RCC cells to TRAIL-induced apoptosis, resulting from the downregulation of c-FLIP (cellular FLICE (FADD-like IL-1β-converting enzyme)-inhibitory protein) via a ROS-dependent pathway [123]. Curcumin has also exhibited promising effects against RCC carcinogenesis by inhibition of cell viability and proliferation, together with the induction of cell cycle arrest and apoptosis through modulation of the PI3K/Akt signaling pathway [124]. Curcumin significantly enhanced the apoptotic effect of the mTOR inhibitor NVP-BEZ235 on RCC cells through p53-dependent Bcl-2 (B-cell lymphoma 2) mRNA down-regulation and impairment of Mcl-1 (myeloid cell leukemia-1) [125]. In a different approach, curcumin enhanced the radiosensitivity of RCC cells by suppressing NF-κB signaling pathway, revealing its potential to be used in combination with radiotherapy of RCC [126].

Other less explored polyphenols have revealed potential effects against RCC carcinogenesis. These include the flavonol conjugate rutin (also known as quercetin-3-rutinoside), which reduced cancer cell viability [127]; kaempferol, also a flavonol, which inhibited RCC cells migration and invasion [128]; and the flavone chrysin that exhibited chemopreventive properties in an in vivo model of chemically induced kidney carcinogenesis [129].

### 2.5. Drug-Induced Nephrotoxicity

In addition to the above-mentioned conditions, nephrotoxicity can also be a consequence of medications’ side effects, as the kidney plays a pivotal role in the detoxification and excretion of toxic metabolites and drugs. Approximately 20% of nephrotoxicity is estimated to be induced by drugs [130]. Cisplatin and gentamicin are two representative examples of clinically relevant nephrotoxic drugs.

Cisplatin is a chemotherapeutic drug widely used against a variety of solid tumors, whose clinical use is limited by the occurrence of nephrotoxic side effects. Cisplatin-induced nephrotoxicity affects mainly the renal tubules and vasculature, and acute tubular necrosis is the most prevalent manifestation [131,132]. Cisplatin accumulates in the proximal tubular epithelial cells and induces oxidative stress, inflammation, vascular injury, and stimulation of apoptosis, thus promoting renal tissue damage [131]. While there is no effective intervention to prevent cisplatin nephrotoxicity in cancer patients to date, many preclinical studies have explored potential protective strategies [131], including the use of polyphenols [132]. Resveratrol ameliorated cisplatin nephrotoxicity in different in vitro and in vivo models, including immortalized proximal tubular epithelial cells from mice [133], rat renal cortical slices [134], mice [133], rats [135,136,137], and rabbits [137]. The mechanisms of protection described for this polyphenol include alterations in apoptosis-associated proteins [133,135], protection against oxidative stress [136,138], inhibition of inflammatory cell infiltrates [136], and activation of SIRT1 [133]. Furthermore, resveratrol protected against cisplatin nephrotoxicity through a pharmacokinetics mechanism, by decreasing its urine concentration and kidney accumulation [137]. Quercetin suppressed the serum creatinine (SCr), indoxyl sulfate, and blood urea nitrogen (BUN) levels in cisplatin-treated rats [139]. Protective effects of this flavonoid were also observed in cultured renal tubular cells [140]. Myricitrin, has been shown to reduce the apoptosis and ROS increase induced by cisplatin in KH-2 cells. In mice, myricitrin reduced the levels of SCr and BUN and protected from morphological alterations and apoptosis induced by cisplatin [141]. EGCG has shown renoprotective effects in rats and mice treated with cisplatin [142,143,144,145]. The proposed mechanisms of protection include the decrease in malondialdehyde (MDA) levels [144,145], restoration of reduced glutathione levels [144,145], inhibition of ER stress-induced apoptosis [143], and regulation of the expression of apoptosis-related proteins (Fas-L, Bax, and Bcl-2) [142]. Another green tea polyphenol, epicatechin gallate (ECG), prevented cisplatin-induced oxidative stress, inflammation, and apoptosis by downregulating the MAPK pathway, leading to improved renal function in cisplatin-treated rats [146]. Treatment with curcumin has shown beneficial effects in several rodent models of cisplatin renal injury [147,148,149,150,151,152,153]. The renoprotective mechanisms of this polyphenol included the prevention of oxidative stress [147,149,150,153] and mitochondrial alterations [147,151], anti-inflammatory effects [148,152], increase in SIRT proteins [151,153], and prevention of cisplatin-induced decrease in tight and adherens junctions [150]. Different phenolic acids have also demonstrated protective effects against cisplatin nephrotoxicity. Ellagic acid has shown beneficial effects in rats [154] and also in colon tumor-bearing mice [155]. In mice, tannic acid, a naturally occurring plant polyphenol that can be found in several plants, counteracted cisplatin nephrotoxicity, by decreasing oxidative stress and DNA damage [156]. Salvianolic acid C protected against cisplatin-induced AKI through attenuation of inflammation, oxidative stress, and apoptosis and by activation of the CaMKK-AMPK-SIRT1-associated signaling pathway in mice [157]. In in vitro models of renal epithelial cells, honokiol counteracted cisplatin-induced cell damage. Besides the decrease of oxidative stress, honokiol promoted the polymerization of actin and tubulin cytoskeleton and stabilized cellular tight junctions, preserving epithelial cell polarity [158]. Nanosized liposomes containing honokiol have also shown to mitigate cisplatin-induced chronic kidney injury in mice [159]. Punicalagin [160] and hydroxytyrosol also prevented cisplatin nephrotoxicity by attenuating inflammation, oxidative stress, and apoptosis [161].

Nephrotoxicity is also a limiting factor for the clinical use of aminoglycoside antibiotics, such as gentamicin. Similar to cisplatin, gentamicin renal damage occurs mainly in the renal tubule due to inflammatory and oxidative mechanisms. Polyphenols are among the compounds that have been studied as potential protectors against gentamicin nephrotoxicity [162]. Resveratrol is the most studied polyphenol in this context. Resveratrol protected against gentamicin nephrotoxicity through antioxidant mechanisms in rat models [163,164]. In mice, resveratrol has shown protective effects against gentamicin-induced EMT in the kidney via inhibition of oxidative stress and TGF-ß/Smad pathway [165]. Morales et al. have proposed that the protective effect of resveratrol against the reduction in kidney function could also be mediated by inhibition of gentamicin-induced mesangial cell contraction [166]. Negrette-Guzmán et al. have shown that curcumin can attenuate gentamicin renal toxicity through modulation of redox pathways and mitochondrial alterations, in vitro and in a rat model [167]. Sinapic acid was also shown to mitigate gentamicin-induced renal injury by downregulating oxidative/nitrosative stress, inflammation, and apoptosis, in rats [168].

Although cisplatin and gentamicin are the two most relevant nephrotoxic drugs, there are also studies considering the impact of other drugs on kidney diseases. The combination of resveratrol and quercetin protected against alterations of the glomerulus ultrastructure and reduced the blood levels of urea and creatinine and biomarkers of inflammation and oxidative stress in a rat model of renal failure induced by acetaminophen [169]. Curcumin demonstrated protective effects on cyclosporine A-induced renal fibrosis in vitro and in a mice model [9]. Radiographic contrast agents are a class of drugs that could also induce kidney toxicity and are a common cause of drug-induced AKI [18]. Salvianolic acid B, one of the principal components of the root of *Salvia miltiorrhiza* used in traditional Chinese medicine, significantly attenuated elevations in serum renal injury markers and histological changes, reduced apoptosis, and lowered the levels of renal oxidative stress in an AKI rat model induced by iohexol, a radiographic contrast agent [170].

Although these studies shown promising results, some of the mentioned polyphenols suffer from low bioavailability and unfavorable pharmacokinetics/pharmacodynamics profiles, in particular, large unmetabolized polyphenols found directly in food sources. For this reason, the biological activities determined in vitro have been a matter of great discussion. Resveratrol, a major compound investigated in the context of renal maladies, is a good example. Despite its broad pharmacological activities towards renoprotection, because resveratrol is poorly bioavailable [171,172], justifications for evaluating direct effects on kidney tissues from a nutritional perspective are not convincing. Regarding in vivo studies using oral administration of polyphenols, it should be noted that the renal protective effect could be mediated by specific metabolites rather than parent compounds. The pharmacokinetics of polyphenols must be thus considered for mechanistic conclusions. To overcome such limitations and to advance the state of the art knowledge on the protective role of polyphenols against nephropathies, it is imperative to focus the investigation efforts on the circulating polyphenol metabolites as the molecules reaching target tissues and exerting potential protective actions, as presented in the next section.

## 3. Polyphenols Bioavailability and Their Relevant Metabolites

**Figure 1:**
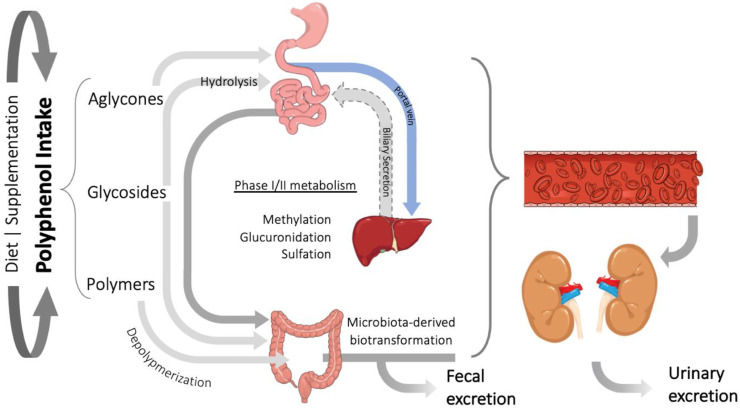
The journey of dietary polyphenols in the human body. Some polyphenols are absorbed in the small intestine, after deglycosylation. There and at the liver, phenolic compounds undergo phase I and II metabolism. From the liver, they are redirected to the small intestine, through biliary secretion and then absorbed into the systemic circulation. Parent polyphenols and metabolites that are not absorbed upstream reach the colon and undergo microbiota metabolism before entering the bloodstream. Polyphenol metabolites in circulation reach target organs and tissues, where they exert their biological activity and are ultimately excreted in the urine. (Figure created in the Mind the Graph platform, available at www.mindthegraph.com, accessed on 23 October 2021).

The role of polyphenols in health promotion has gained wide interest from the scientific community in the past few decades. However, only few studies have considered the metabolism and bioavailability of these compounds in vivo, which are crucial aspects to understand the beneficial contribution of polyphenols for human health [173,174]. The journey of dietary polyphenols after human intake is relatively complex. In nature, most polyphenols occur as polymers or in association with other molecules, such as carbohydrates and organic acids. The absorption of these molecules is dependent on the catabolic reactions releasing the phenolic structure, or aglycone, from its associated structures [175,176,177]. While some polyphenols can be directly absorbed in the stomach, such as anthocyanins, some isoflavones, and quercetin, most of them are absorbed in the small intestine and pass through a series of chemical alterations conferring them distinct fates [178,179], as summarized in Figure 1.

In the small intestine, lactase phlorizin hydrolase (LPH) and cytosolic β-glucosidase (CBG) enzymes catalyze the hydrolysis of glucoside conjugates, promoting the cellular uptake of polyphenol aglycones by enterocytes and their passage to the liver through the portal vein [176,180,181]. These phenolic structures undergo different conjugation reactions such as methylation, glucuronidation, or sulfation by phase II enzymatic metabolism in the enterocytes and hepatocytes [181]. Polyphenol conjugates re-enter the intestine via biliary secretion and are directed to the colon, where extensive microbiota-derived biotransformation generates different low molecular weight structures that will reach the bloodstream [178,181,182]. Interestingly, those low-molecular-weight compounds generated from polyphenols through hepatic and microbiota metabolism may reach circulation at considerably higher concentrations than their parent compounds [174,178,179]. While the blood concentration of parent polyphenols is usually in the nanomolar range, some metabolites reached concentrations of over 25 μM in human volunteers [183]. Once in circulation, polyphenol metabolites are known to be distributed across a wide variety of tissues and have inclusively been shown to cross the blood–brain barrier. Previous studies carried out in rats have also demonstrated considerable amounts of polyphenol metabolites in kidney tissue, after ingestion of a berries-rich diet [101,102,184]. Polyphenol metabolites interact with target tissues and cells, where they can exert relevant biological activities. Finally, polyphenol metabolites are excreted via urinary clearance [175,178,183,185]. The large majority of the known circulating polyphenol metabolites derive from a group of structurally diverse parent compounds (e.g., chlorogenic acids, flavanols, proanthocyanidins, theaflavins, and thearubigins) that undergo modifications converging to formation of aromatic/phenolic acids with hydroxyls substituents whereas fewer are associated with a unique circulating polyphenol metabolite (e.g., urolithins from ellagitannins, S-equol from isoflavones). In the frame of this review, we only consider the general polyphenol metabolites that reach the circulation at higher concentrations compared to their parent polyphenol compound counterparts and are thus of much more biological relevance. Unique circulating polyphenol metabolites due to their lower nutritional relevance were excluded.

**Figure 2:**
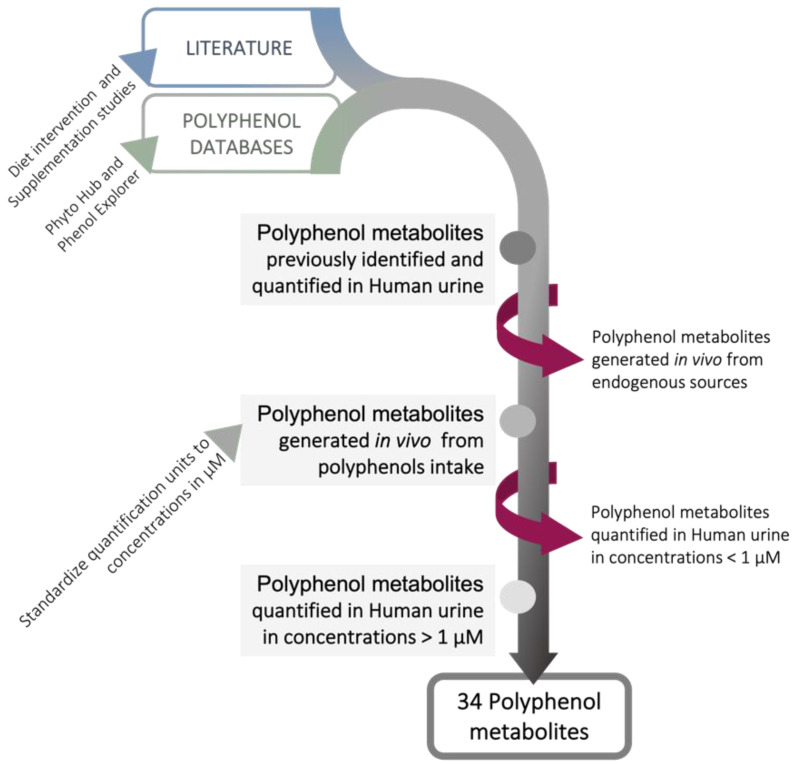
Selection of physiologically relevant polyphenol metabolites for kidney protection. We collected available data from both the literature on dietary and supplementation intervention studies as well as from two polyphenol databases, Phyto Hub and Phenol-Explorer. These data allowed the identification of the most nutritionally relevant circulating polyphenol metabolites previously detected and quantified in human urine. We then estimated the maximum urinary concentrations in µM. After exclusion of phenolic metabolites that are known to be also generated from endogenous sources, as well as those identified in human urine in concentrations < 1 µM, this approach led us to a selection of 34 polyphenol metabolites.

When the aim is to evaluate the effects of polyphenols on kidney protection, it is important to identify those compounds that actually contact kidney cells. However, compounds levels in human kidney tissues are rarely available, since this could only be assessed by highly invasive approaches. Urinary concentrations of compounds could thus provide a relative estimation on the compounds and concentrations contacting kidney cells. However, it must be mentioned that the estimation of kidney tissue levels based on urinary concentrations has some limitations. The overall excretion of xenobiotics is a result of the physiological events occurring in nephrons, glomerular filtration, active tubular secretion and reabsorption, which depend on the physical-chemical properties of the compounds. During their journey across the nephron, xenobiotics may accumulate within the tubular cells. The extent of this accumulation will depend on the efficiency of compounds uptake during secretion, intracellular binding to cytosolic proteins, sequestration within cellular organelles, intracellular trafficking, and efficiency of efflux into the lumen [186]. In addition, kidney cells may also play a role in metabolism, both through phase I and phase II biotransformation reactions [186]. Due to these phenomena, the compounds and concentrations detected in urine may not exactly reflect the renal cells exposure. Nevertheless, the polyphenol metabolite profiling in urine provides a basis to obtain a rough estimation of the range of concentrations that occur in human kidney tissues, that otherwise would not be available. For this purpose, we have performed an extensive survey on the available data regarding the identification and quantification of polyphenol metabolites in human urine, as illustrated in Figure 2. 

Data on the compounds detected in urine were collected from published literature and from the databases Phenol-Explorer [187] and PhytoHub [188]. Most of the studies were carried out in human healthy volunteers and the urinary metabolites were analyzed through different techniques, such as GC-MS, LC-MS, and HPLC. Parent compounds and metabolites formed from sources other than polyphenols intake were excluded. To allow quantitative comparison between the different studies, the maximum urinary concentration achieved in each study was calculated. For this calculation, a mean urine volume of 1900 mL/day and a daily creatinine clearance of 92.4 mg/dL were considered [189,190]. Thirty-four metabolites were identified in human urine, in maximum concentrations higher than 1 µM. Those metabolites are detailed in Table 1. It should be mentioned that the polyphenol metabolites selected for Table 1 could be formed from multiple parent compounds and food sources. However, only the experimental conditions in which the maximum urinary concentrations were detected for each metabolite are shown.

Although the available human studies dealing with the pharmacokinetics of polyphenols were essentially carried out in healthy individuals, it is known that kidney diseases could alter the in vivo fate of many compounds. This should be taken into account when the bioavailability of polyphenols and their metabolites are studied [203]. Moreover, intestinal microbial dysbiosis, with primary source or secondary to kidney diseases, such as CKD, may also affect the bioavailability of many compounds [204]. Thus, to better estimate the bioavailability of polyphenols and their metabolites in individuals with kidney disease and to consider their beneficial role, all those physiopathological modifications should be considered in experimental studies.

## 4. Conclusions

The studies compiled herein exemplify the plethora of potential bioactivities exerted by polyphenols against renal disease complications. As disclosed for each kidney pathology, the biological effects of polyphenols have been associated with the modulation of specific signaling cascades including those involved in oxidative stress responses, anti-inflammation processes, and apoptosis. Although there is increasing evidence that polyphenols afford great potential in renal disease therapy, the evidence presented here should be considered with caution before its clinical translation, particularly due to the unfavorable pharmacokinetics and/or pharmacodynamics profiles of the parent compounds. This aspect is particularly critical when in vitro studies are concerned. From a nutritional perspective, a limitation of most of the previous studies lies in the exploration of low-bioavailable compounds and, on rare occasions, circulating polyphenol metabolites at concentrations that are not achievable in the human circulation and target tissues. Further research based on well-designed clinical trials is needed to unravel the efficacy of these compounds towards the mitigation of kidney diseases. At this point, it is important to emphasize that in a physiological context, circulating polyphenol metabolites are prone to reach target cells in higher concentrations than the parent compounds. Thus, understanding the effects of circulating polyphenol metabolites on kidney pathologies is essential to explore the role of dietary polyphenol intake from a physiologically relevant perspective.

## Figures and Tables

**Table 1:** Polyphenol metabolites previously identified in human urine, resulting from polyphenol intake, quantified in concentrations above 1 µM. For each metabolite, only the study in which the maximum concentration was detected, and the respective experimental conditions are shown.

Polyphenol Metabolites	Max. Concentration in Urine (µM) *	Experimental Conditions		Reference
		Polyphenol Source	Ingested Dose	
4′-Hydroxyphenylacetic acid	406.8	Green tea	300 mL (single dose)	[191]
Phenylacetic acid	153.3	Cocoa powder in whole milk	40 g (single dose)	[192]
3′-Hydroxyphenylacetic acid	136.3	Quercetin 3-*O*-rutinoside	440 mg/24 h (7 days)	[193]
3,4-Dihydroxybenzoic acid (Protocatechuic acid)	64.8	Quercetin	200 mg (single dose)	[194]
4′-Hydroxy-3′-methoxyphenylacetic acid (homovanillic acid)	54.2	Quercetin 3-*O*-rutinoside	440 mg/24 h (7 days)	[193]
4-Hydroxy-3-methoxybenzoic acid (Vanillic acid)	53.6	5-caffeoylquinic acid	2 g/24 h (7 days)	[193]
3-(3′,4′-Dihydroxyphenyl)propanoic acid (Dihydrocaffeic acid)	51.1	5-caffeoylquinic acid	2 g/24 h (7 days)	[193]
4′-Hydroxy-3′-methoxycinnamic acid (Ferulic acid)	37.4	5-caffeoylquinic acid	2 g/24 h (7 days)	[193]
3-Hydroxybenzoic acid	35.8	Black tea solids	4 g/24 h (7 days)	[193]
Benzoic acid	31.5	Quercetin	200 mg (single dose)	[194]
3′,4′-Dihydroxycinnamic acid (Caffeic acid)	29.5	Tablets of perilla extract	1 tablet (single dose)	[195]
3′-Hydroxycinnamic acid	22.5	5-caffeoylquinic acid	2 g/24 h (7 days)	[193]
2,3-Dihydroxybenzoic acid	22.5	Freeze-dried blueberry powder	22 g (during 24 h)	[196]
4-Hydroxybenzoic acid	19.7	Cocoa powder in skimmed milk	40 g/24 h (4 weeks)	[197]
(E)-3-(4′-Hydroxy-3′,5′-dimethoxyphenyl)prop-2-enoic acid (Sinapic acid)	13.7	Quercetin 3-*O*-rutinoside	440 mg/24 h (7 days)	[193]
4-Hydroxy-3,5-dimethoxybenzoic acid (Syringic acid)	7.2	5-caffeoylquinic acid	2 g/24 h (7 days)	[193]
4-Ethylphenol	7.0	Quercetin	200 mg (single dose)	[194]
3′-Methoxycinnamic acid-4′-sulfate (Ferulic acid-sulfate)	6.4	Freeze-dried blueberry powder	22 g (during 24 h)	[196]
2-(3′,4′-Dihydroxyphenyl)ethanol (Hydroxytyrosol)	6.1	Tyrosol (140 μg/mL)	50 mL (single dose)	[198]
1,3,5-Trimethoxybenzene(Phloroglucinol)	5.9	(−)-epicatechin	200 mg (single dose)	[194]
3-Methoxybenzoic acid-4-glucuronide (Vanillic acid-glucuronide)	5.7	Red grape pomace aqueous extract	250 mL (single dose)	[199]
2′-Hydroxyhippuric acid	5.1	Black tea solids	4 g/24 h (7 days)	[193]
4-(2′-Hydroxyethyl)-2-methoxyphenol	4.4	Olive oil with phenol extract	50 mL of 1950 mg/L total phenols extract (single dose)	[200]
3,4,5-Trihydroxybenzoic acid (Gallic acid)	3.7	Black tea solids	4 g/24 h (7 days)	[193]
Phenol-2-sulfate (Catechol-sulfate)	3.1	Cranberry juice	450 mL (single dose)	[201]
3′-Methoxycinnamic acid-4′-glucuronide (Ferulic acid-glucuronide)	2.8	Freeze-dried blueberry powder	22 g (during 24 h)	[196]
4′-Hydroxycinnamic acid (p-coumaric acid)	2.2	5-caffeoylquinic acid	2 g/24 h (7 days)	[193]
4-Methylcatechol-*O*-sulfate	2.1	Freeze-dried blueberry powder	22 g (during 24 h)	[196]
3-(4′-Hydroxyphenyl)propanoic acid-3′-sulfate	2.1	Freeze-dried blueberry powder	22 g (during 24 h)	[196]
3-(3′-Methoxyphenyl)propanoic acid-4′-sulfate	1.8	Freeze-dried blueberry powder	22 g (during 24 h)	[196]
3-Methoxybenzoic acid-4-sulfate (Vanillic acid-4-sulfate)	1.7	Cyanidin-3-glucoside	500 mg (single dose)	[202]
3-Hydroxybenzoic acid-4-sulfate (Protocatechuic acid-4-sulfate	1.2	Cyanidin-3-glucoside	500 mg (single dose)	[202]
Benzene-1,2-diol (Catechol)	1.2	Green tea	300 mL (single dose)	[191]
4-Hydroxybenzoic acid-3-sulfate (Protocatechuic acid-3-sulfate)	1.1	Cyanidin-3-glucoside	500 mg (single dose)	[202]

The maximum urinary concentration of polyphenol metabolites in µM was calculated using quantifications from each study, considering a mean urine volume of 1900 mL/day and a daily creatinine clearance of 92.4 mg/dL [189,190].

## Data Availability

Data is contained within the article.

## References

[1] Bikbov B., Purcell C.A., Levey A.S., Smith M., Abdoli A., Abebe M., Adebayo O.M., Afarideh M., Agarwal S.K., Agudelo-Botero M. (2020). Global, regional, and national burden of chronic kidney disease, 1990–2017: A systematic analysis for the Global Burden of Disease Study 2017. Lancet.

[2] Saran R., Robinson B., Abbott K.C., Bragg-Gresham J., Chen X., Gipson D., Gu H., Hirth R.A., Hutton D., Jin Y. (2020). US Renal Data System 2019 Annual Data Report: Epidemiology of Kidney Disease in the United States. Am. J. Kidney Dis..

[3] Levey A.S., Titan S.M., Powe N.R., Coresh J., Inker L.A. (2020). Kidney disease, race, and gfr estimation. Clin. J. Am. Soc. Nephrol..

[4] Palmer S.C., Maggo J.K., Campbell K.L., Craig J.C., Johnson D.W., Sutanto B., Ruospo M., Tong A., Strippoli G.F.M. (2017). Dietary interventions for adults with chronic kidney disease. Cochrane Database Syst. Rev..

[5] Zhang S., Jia Z., Yan Z., Yang J. (2017). Consumption of fruits and vegetables and risk of renal cell carcinoma: A meta-analysis of observational studies. Oncotarget.

[6] Noce A., Bocedi A., Campo M., Marrone G., Di Lauro M., Cattani G., Di Daniele N., Romani A. (2020). A pilot study of a natural food supplement as new possible therapeutic approach in chronic kidney disease patients. Pharmaceuticals.

[7] Turki K., Charradi K., Boukhalfa H., Belhaj M., Limam F., Aouani E., Unit H., Unit H., Hospital H.B. (2016). Grape seed powder improves renal failure of chronic kidney disease patients. EXCL1 J..

[8] Lin Y.F., Lee Y.H., Hsu Y.H., Chen Y.J., Lin Y.F., Cheng F.Y., Chiu H.W. (2017). Resveratrol-loaded nanoparticles conjugated with kidney injury molecule-1 as a drug delivery system for potential use in chronic kidney disease. Nanomedicine.

[9] Hu Y., Mou L., Yang F., Tu H., Lin W. (2016). Curcumin attenuates cyclosporine A-induced renal fibrosis by inhibiting hypermethylation of the klotho promoter. Mol. Med. Rep..

[10] Daenen K., Andries A., Mekahli D., Van Schepdael A., Jouret F., Bammens B. (2019). Oxidative stress in chronic kidney disease. Pediatr. Nephrol..

[11] Pavlakou P., Liakopoulos V., Eleftheriadis T., Mitsis M., Dounousi E. (2017). Oxidative Stress and Acute Kidney Injury in Critical Illness: Pathophysiologic Mechanisms—Biomarkers—Interventions, and Future Perspectives. Oxid. Med. Cell. Longev..

[12] Martínez-Klimova E., Aparicio-Trejo O.E., Gómez-Sierra T., Jiménez-Uribe A.P., Bellido B., Pedraza-Chaverri J. (2020). Mitochondrial dysfunction and endoplasmic reticulum stress in the promotion of fibrosis in obstructive nephropathy induced by unilateral ureteral obstruction. BioFactors.

[13] Podkowińska A., Formanowicz D. (2020). Chronic Kidney Disease as Oxidative Stress- and Inflammatory-Mediated Cardiovascular Disease. Antioxidants.

[14] Ling X.C., Kuo K.-L. (2018). Oxidative stress in chronic kidney disease. Ren. Replace. Ther..

[15] Kalantar-Zadeh K., Jafar T.H., Nitsch D., Neuen B.L., Perkovic V. (2021). Chronic kidney disease. Lancet.

[16] Li Y.R., Trush M. (2016). Defining ROS in Biology and Medicine. React. Oxyg. Species.

[17] Ronco C., Bellomo R., Kellum J.A. (2019). Acute kidney injury. Lancet.

[18] Kellum J.A., Romagnani P., Ashuntantang G., Ronco C., Zarbock A., Anders H.J. (2021). Acute kidney injury. Nat. Rev. Dis. Prim..

[19] Levey A.S., James M.T. (2017). Annals graphic medicine—The problem list. Ann. Intern. Med..

[20] Huang Y.T., Chen Y.Y., Lai Y.H., Cheng C.C., Lin T.C., Su Y.S., Liu C.H., Lai P.C. (2016). Resveratrol alleviates the cytotoxicity induced by the radiocontrast agent, ioxitalamate, by reducing the production of reactive oxygen species in HK-2 human renal proximal tubule epithelial cells in vitro. Int. J. Mol. Med..

[21] Wang Y., Feng F., Liu M., Xue J., Huang H. (2018). Resveratrol ameliorates sepsis-induced acute kidney injury in a pediatric rat model via Nrf2 signaling pathway. Exp. Ther. Med..

[22] Wang N., Mao L., Yang L., Zou J., Liu K., Liu M., Zhang H., Xiao X., Wang K. (2017). Resveratrol protects against early polymicrobial sepsis-induced acute kidney injury through inhibiting endoplasmic reticulum stress-activated NF-κB pathway. Oncotarget.

[23] Chen L., Yang S., Zumbrun E.E., Guan H., Nagarkatti P.S., Nagarkatti M. (2015). Resveratrol attenuates lipopolysaccharide-induced acute kidney injury by suppressing inflammation driven by macrophages. Mol. Nutr. Food Res..

[24] Luo C.J., Luo F., Bu Q.D., Jiang W., Zhang W., Liu X.M., Che L., Luan H., Zhang H., Ma R.X. (2020). Protective effects of resveratrol on acute kidney injury in rats with sepsis. Biomed. Pap..

[25] Holthoff J.H., Wang Z., Seely K.A., Gokden N., Mayeux P.R. (2012). Resveratrol improves renal microcirculation, protects the tubular epithelium, and prolongs survival in a mouse model of sepsis-induced acute kidney injury. Kidney Int..

[26] Xu S., Gao Y., Zhang Q., Wei S., Chen Z., Dai X., Zeng Z., Zhao K.S. (2016). SIRT1/3 activation by Resveratrol attenuates acute kidney injury in a septic rat model. Oxid. Med. Cell. Longev..

[27] Gao Y., Zeng Z., Li T., Xu S., Wang X., Chen Z., Lin C. (2015). Polydatin inhibits mitochondrial dysfunction in the renal tubular epithelial cells of a rat model of sepsis-induced acute kidney injury. Anesth. Analg..

[28] Singh J.P., Singh A.P., Bhatti R. (2014). Explicit role of peroxisome proliferator-activated receptor gamma in gallic acid-mediated protection against ischemia-reperfusion-induced acute kidney injury in rats. J. Surg. Res..

[29] Bao G.H., Xu J., Hu F.L., Wan X.C., Deng S.X., Barasch J. (2013). EGCG inhibit chemical reactivity of iron through forming an Ngal-EGCG-iron complex. BioMetals.

[30] Twal M., Kiefer P., Salameh A., Schnabel J., Ossmann S., Von Salisch S., Krämer K., Sobiraj A., Kostelka M., Mohr F.W. (2013). Reno-protective effects of epigallocatechingallate in a small piglet model of extracorporeal circulation. Pharmacol. Res..

[31] Funamoto M., Masumoto H., Takaori K., Taki T., Setozaki S., Yamazaki K., Minakata K., Ikeda T., Hyon S.H., Sakata R. (2016). Green Tea Polyphenol Prevents Diabetic Rats from Acute Kidney Injury after Cardiopulmonary Bypass Presented at the American Heart Association Scientific Session, Chicago, IL, Nov 15–19, 2014. Ann. Thorac. Surg..

[32] Kakuta Y., Okumi M., Isaka Y., Tsutahara K., Abe T., Yazawa K., Ichimaru N., Matsumura K., Hyon S.H., Takahara S. (2011). Epigallocatechin-3-gallate protects kidneys from ischemia reperfusion injury by HO-1 upregulation and inhibition of macrophage infiltration. Transpl. Int..

[33] Fan Y., Chen H., Peng H., Huang F., Zhong J., Zhou J. (2017). Molecular mechanisms of curcumin renoprotection in experimental acute renal injury. Front. Pharmacol..

[34] Liu Q., Liang X., Liang M., Qin R., Qin F., Wang X. (2020). Ellagic Acid Ameliorates Renal Ischemic-Reperfusion Injury Through NOX4/JAK/STAT Signaling Pathway. Inflammation.

[35] Xia S., Lin H., Liu H., Lu Z., Wang H., Fan S., Li N. (2019). Honokiol Attenuates Sepsis-Associated Acute Kidney Injury via the Inhibition of Oxidative Stress and Inflammation. Inflammation.

[36] Webster A.C., Nagler E.V., Morton R.L., Masson P. (2017). Chronic Kidney Disease. Lancet.

[37] Romagnani P., Remuzzi G., Glassock R., Levin A., Jager K.J., Tonelli M., Massy Z., Wanner C., Anders H.J. (2017). Chronic kidney disease. Nat. Rev. Dis. Prim..

[38] Li P., Song X., Zhang D., Guo N., Wu C., Chen K., Liu Y., Yuan L., Chen X., Huang X. (2020). Resveratrol improves left ventricular remodeling in chronic kidney disease via Sirt1-mediated regulation of FoxO1 activity and MnSOD expression. BioFactors.

[39] Liang J., Tian S., Han J., Xiong P. (2014). Resveratrol as a therapeutic agent for renal fibrosis induced by unilateral ureteral obstruction. Ren. Fail..

[40] Hui Y., Lu M., Han Y., Zhou H., Liu W., Li L., Jin R. (2017). Resveratrol improves mitochondrial function in the remnant kidney from 5/6 nephrectomized rats. Acta Histochem..

[41] Sun L.J., Sun Y.N., Chen S.J., Liu S., Jiang G.R. (2017). Resveratrol attenuates skeletal muscle atrophy induced by chronic kidney disease via MuRF1 signaling pathway. Biochem. Biophys. Res. Commun..

[42] Wang Y., Wang B., Du F., Su X., Sun G., Zhou G., Bian X., Liu N. (2015). Epigallocatechin-3-Gallate Attenuates Oxidative Stress and Inflammation in Obstructive Nephropathy via NF-κB and Nrf2/HO-1 Signalling Pathway Regulation. Basic Clin. Pharmacol. Toxicol..

[43] Kanlaya R., Thongboonkerd V. (2019). Molecular Mechanisms of Epigallocatechin-3-Gallate for Prevention of Chronic Kidney Disease and Renal Fibrosis: Preclinical Evidence. Curr. Dev. Nutr..

[44] Hongtao C., Youling F., Fang H., Huihua P., Jiying Z., Jun Z. (2018). Curcumin alleviates ischemia reperfusion-induced late kidney fibrosis through the APPL1/Akt signaling pathway. J. Cell. Physiol..

[45] Wang J.H., Zhang H.F., Wang J.H., Wang Y.L., Gao C., Gu Y.T., Huang J., Zhang Z. (2019). Salvianolic Acid A Protects the Kidney against Oxidative Stress by Activating the Akt/GSK-3 β/Nrf2 Signaling Pathway and Inhibiting the NF- B Signaling Pathway in 5/6 Nephrectomized Rats. Oxid. Med. Cell. Longev..

[46] Lin Y.C., Chang Y.H., Yang S.Y., Wu K.D., Chu T.S. (2018). Update of pathophysiology and management of diabetic kidney disease. J. Formos. Med. Assoc..

[47] Sugahara M., Pak W.L.W., Tanaka T., Tang S.C.W., Nangaku M. (2021). Update on diagnosis, pathophysiology, and management of diabetic kidney disease. Nephrology.

[48] Pani A., Baratta F., Pastori D., Coronati M., Scaglione F., del Ben M. (2021). Prevention and management of type II diabetes chronic complications: The role of polyphenols (Mini-Review). Curr. Med. Chem..

[49] Den Hartogh D.J., Tsiani E. (2019). Health benefits of resveratrol in kidney disease: Evidence from in vitro and in vivo studies. Nutrients.

[50] Gowd V., Kang Q., Wang Q., Wang Q., Chen F., Cheng K.W. (2020). Resveratrol: Evidence for Its Nephroprotective Effect in Diabetic Nephropathy. Adv. Nutr..

[51] Li K.X., Ji M.J., Sun H.J. (2021). An updated pharmacological insight of resveratrol in the treatment of diabetic nephropathy. Gene.

[52] Hashemzaei M., Tabrizian K., Alizadeh Z., Pasandideh S., Rezaee R., Mamoulakis C., Tsatsakis A., Skaperda Z., Kouretas D., Shahraki J. (2020). Resveratrol, curcumin and gallic acid attenuate glyoxal-induced damage to rat renal cells. Toxicol. Rep..

[53] Zhang J., Dong X.J., Ding M.R., You C.Y., Lin X., Wang Y., Wu M.J.Y., Xu G.F., Wang G.D. (2020). Resveratrol decreases high glucose-induced apoptosis in renal tubular cells via suppressing endoplasmic reticulum stress. Mol. Med. Rep..

[54] Wang F., Li R., Zhao L., Ma S., Qin G. (2020). Resveratrol ameliorates renal damage by inhibiting oxidative stress-mediated apoptosis of podocytes in diabetic nephropathy. Eur. J. Pharmacol..

[55] Wang Y., Wang B., Qi X., Zhang X., Ren K. (2019). Resveratrol Protects Against Post-Contrast Acute Kidney Injury in Rabbits with Diabetic Nephropathy. Front. Pharmacol..

[56] Xian Y., Gao Y., Lv W., Ma X., Hu J., Chi J., Wang W., Wang Y. (2020). Resveratrol prevents diabetic nephropathy by reducing chronic inflammation and improving the blood glucose memory effect in non-obese diabetic mice. Naunyn-Schmiedebergs Arch. Pharmacol..

[57] Gong W., Li J., Chen W., Feng F., Deng Y. (2020). Resveratrol inhibits lipopolysaccharide-induced extracellular matrix accumulation and inflammation in rat glomerular mesangial cells by sphk1/s1p2/nf-κb pathway. Diabetes Metab. Syndr. Obes. Targets Ther..

[58] Peng X., Su H., Liang D., Li J., Ting W.J., Liao S.C., Huang C.Y. (2019). Ramipril and resveratrol co-treatment attenuates RhoA/ROCK pathway-regulated early-stage diabetic nephropathy-associated glomerulosclerosis in streptozotocin-induced diabetic rats. Environ. Toxicol..

[59] Xie X., Peng J., Huang K., Huang J., Shen X., Liu P., Huang H. (2012). Polydatin ameliorates experimental diabetes-induced fibronectin through inhibiting the activation of NF-κB signaling pathway in rat glomerular mesangial cells. Mol. Cell. Endocrinol..

[60] Huang K., Chen C., Hao J., Huang J., Wang S., Liu P., Huang H. (2015). Polydatin promotes Nrf2-ARE anti-oxidative pathway through activating Sirt1 to resist AGEs-induced upregulation of fibronetin and transforming growth factor-β1 in rat glomerular messangial cells. Mol. Cell. Endocrinol..

[61] Gong W., Li J., Chen Z., Huang J., Chen Q., Cai W., Liu P., Huang H. (2017). Polydatin promotes Nrf2-ARE anti-oxidative pathway through activating CKIP-1 to resist HG-induced up-regulation of FN and ICAM-1 in GMCs and diabetic mice kidneys. Free Radic. Biol. Med..

[62] El-Hameed A., Abeer M. (2020). Polydatin-loaded chitosan nanoparticles ameliorates early diabetic nephropathy by attenuating oxidative stress and inflammatory responses in streptozotocin-induced diabetic rat. J. Diabetes Metab. Disord..

[63] Ni Z., Tao L., Xiaohui X., Zelin Z., Jiangang L., Zhao S., Weikang H., Hongchao X., Qiujing W., Xin L. (2017). Polydatin impairs mitochondria fitness and ameliorates podocyte injury by suppressing Drp1 expression. J. Cell. Physiol..

[64] Chen Z.Q., Sun X.H., Li X.J., Xu Z.C., Yang Y., Lin Z.Y., Xiao H.M., Zhang M., Quan S.J., Huang H.Q. (2020). Polydatin attenuates renal fibrosis in diabetic mice through regulating the Cx32-Nox4 signaling pathway. Acta Pharmacol. Sin..

[65] An X., Zhang Y., Cao Y., Chen J., Qin H., Yang L. (2020). Punicalagin protects diabetic nephropathy by inhibiting pyroptosis based on TXNIP/NLRP3 pathway. Nutrients.

[66] Zheng H.X., Qi S.S., He J., Hu C.Y., Han H., Jiang H., Li X.S. (2020). Cyanidin-3-glucoside from Black Rice Ameliorates Diabetic Nephropathy via Reducing Blood Glucose, Suppressing Oxidative Stress and Inflammation, and Regulating Transforming Growth Factor β1/Smad Expression. J. Agric. Food Chem..

[67] Qin Y., Zhai Q., Li Y., Cao M., Xu Y., Zhao K., Wang T. (2018). Cyanidin-3-*O*-glucoside ameliorates diabetic nephropathy through regulation of glutathione pool. Biomed. Pharmacother..

[68] Wei J., Wu H., Zhang H., Li F., Chen S., Hou B., Shi Y., Zhao L., Duan H. (2018). Anthocyanins inhibit high glucose-induced renal tubular cell apoptosis caused by oxidative stress in db/db mice. Int. J. Mol. Med..

[69] Wang S., Huang Y., Luo G., Yang X., Huang W. (2021). Cyanidin-3-o-glucoside attenuates high glucose–induced podocyte dysfunction by inhibiting apoptosis and promoting autophagy via activation of sirt1/ampk pathway. Can. J. Physiol. Pharmacol..

[70] Lewandowska H., Kalinowska M., Lewandowski W., Stepkowski T.M., Brzóska K. (2016). The role of natural polyphenols in cell signaling and cytoprotection against cancer development. J. Nutr. Biochem..

[71] Ma Y., Chen F., Yang S., Chen B., Shi J. (2018). Protocatechuic acid ameliorates high glucose-induced extracellular matrix accumulation in diabetic nephropathy. Biomed. Pharmacother..

[72] Oza M.J., Kulkarni Y.A. (2019). Formononetin attenuates kidney damage in type 2 diabetic rats. Life Sci..

[73] Xu W.L., Liu S., Li N., Ye L.F., Zha M., Li C.Y., Zhao Y., Pu Q., Bao J.J., Chen X.J. (2021). Quercetin Antagonizes Glucose Fluctuation Induced Renal Injury by Inhibiting Aerobic Glycolysis via HIF-1α/miR-210/ISCU/FeS Pathway. Front. Med..

[74] Du L., Li C., Qian X., Chen Y., Wang L., Yang H., Li X., Li Y., Yin X., Lu Q. (2019). Quercetin inhibited mesangial cell proliferation of early diabetic nephropathy through the Hippo pathway. Pharmacol. Res..

[75] Jiang X., Yu J., Wang X., Ge J., Li N. (2019). Quercetin improves lipid metabolism via SCAP-SREBP2-LDLr signaling pathway in early stage diabetic nephropathy. Diabetes Metab. Syndr. Obes. Targets Ther..

[76] Tang L., Li K., Zhang Y., Li H., Li A., Xu Y., Wei B. (2020). Quercetin liposomes ameliorate streptozotocin-induced diabetic nephropathy in diabetic rats. Sci. Rep..

[77] Tong F., Liu S., Yan B., Li X., Ruan S., Yang S. (2017). Quercetin nanoparticle complex attenuated diabetic nephropathy via regulating the expression level of ICAM-1 on endothelium. Int. J. Nanomed..

[78] Zhou J., Zhang S., Sun X., Lou Y., Bao J., Yu J. (2021). Hyperoside ameliorates diabetic nephropathy induced by STZ via targeting the miR-499–5p/APC axis. J. Pharmacol. Sci..

[79] Ding T., Wang S., Zhang X., Zai W., Fan J., Chen W., Bian Q., Luan J., Shen Y., Zhang Y. (2018). Kidney protection effects of dihydroquercetin on diabetic nephropathy through suppressing ROS and NLRP3 inflammasome. Phytomedicine.

[80] Dua T.K., Joardar S., Chakraborty P., Bhowmick S., Saha A., De Feo V., Dewanjee S. (2021). Myricitrin, a glycosyloxyflavone in myrica esculenta bark ameliorates diabetic nephropathy via improving glycemic status, reducing oxidative stress, and suppressing inflammation. Molecules.

[81] Ahangarpour A., Oroojan A.A., Khorsandi L., Kouchak M., Badavi M. (2019). Antioxidant, anti-apoptotic, and protective effects of myricitrin and its solid lipid nanoparticles on streptozotocin-nicotinamideinduced diabetic nephropathy in type 2 diabetic male mice. Iran. J. Basic Med. Sci..

[82] Kanlaya R., Thongboonkerd V. (2019). Protective Effects of Epigallocatechin-3-Gallate from Green Tea in Various Kidney Diseases. Adv. Nutr..

[83] Mohan T., Velusamy P., Chakrapani L.N., Srinivasan A.K., Singh A., Johnson T., Periandavan K. (2017). Impact of EGCG Supplementation on the Progression of Diabetic Nephropathy in Rats: An Insight into Fibrosis and Apoptosis. J. Agric. Food Chem..

[84] Yoon S.P., Maeng Y.H., Hong R., Lee B.R., Kim C.G., Kim H.L., Chung J.H., Shin B.C. (2014). Protective effects of epigallocatechin gallate (EGCG) on streptozotocin-induced diabetic nephropathy in mice. Acta Histochem..

[85] Mohan T., Narasimhan K.K.S., Ravi D.B., Velusamy P., Chandrasekar N., Chakrapani L.N., Srinivasan A., Karthikeyan P., Kannan P., Tamilarasan B. (2020). Role of Nrf2 dysfunction in the pathogenesis of diabetic nephropathy: Therapeutic prospect of epigallocatechin-3-gallate. Free Radic. Biol. Med..

[86] Hayashi D., Wang L., Ueda S., Yamanoue M., Ashida H., Shirai Y. (2020). The mechanisms of ameliorating effect of a green tea polyphenol on diabetic nephropathy based on diacylglycerol kinase α. Sci. Rep..

[87] Zhu Q.Q., Yang X.Y., Zhang X.J., Yu C.J., Pang Q.Q., Huang Y.W., Wang X.J., Sheng J. (2020). EGCG targeting Notch to attenuate renal fibrosis: Via inhibition of TGFβ/Smad3 signaling pathway activation in streptozotocin-induced diabetic mice. Food Funct..

[88] Xiang C., Xiao X., Jiang B., Zhou M., Zhang Y., Li H., Hu Z. (2017). Epigallocatechin-3-gallate protects from high glucose induced Podocyte apoptosis via suppressing endoplasmic reticulum stress. Mol. Med. Rep..

[89] Álvarez Cilleros D., López-Oliva M.E., Martín M.Á., Ramos S. (2020). (−)-Epicatechin and the colonic metabolite 2,3-dihydroxybenzoic acid protect against high glucose and lipopolysaccharide-induced inflammation in renal proximal tubular cells through NOX-4/p38 signalling. Food Funct..

[90] Bao L., Cai X., Dai X., Ding Y., Jiang Y., Li Y., Zhang Z., Li Y. (2014). Grape seed proanthocyanidin extracts ameliorate podocyte injury by activating peroxisome proliferator-activated receptor-γ coactivator 1α in low-dose streptozotocin-and high-carbohydrate/high-fat diet-induced diabetic rats. Food Funct..

[91] Cai X., Bao L., Ren J., Li Y., Zhang Z. (2016). Grape seed procyanidin B2 protects podocytes from high glucose-induced mitochondrial dysfunction and apoptosis via the AMPK-SIRT1-PGC-1α axis in vitro. Food Funct..

[92] Zhu D., Wang L., Zhou Q., Yan S., Li Z., Sheng J., Zhang W. (2014). (+)-Catechin ameliorates diabetic nephropathy by trapping methylglyoxal in type 2 diabetic mice. Mol. Nutr. Food Res..

[93] Liu H.W., Wei C.C., Chang S.J. (2016). Low-molecular-weight polyphenols protect kidney damage through suppressing NF-κB and modulating mitochondrial biogenesis in diabetic: Db/db mice. Food Funct..

[94] Park C.H., Yokozawa T., Noh J.S. (2014). Oligonol, a low-molecular-weight polyphenol derived from lychee fruit, attenuates diabetes-induced renal damage through the advanced glycation end product-related pathway in db/db mice. J. Nutr..

[95] Park C.H., Noh J.S., Fujii H., Roh S.-S., Song Y.-O., Choi J.S., Chung H.Y., Yokozawa T. (2015). Oligonol, a low-molecular-weight polyphenol derived from lychee fruit, attenuates gluco-lipotoxicity-mediated renal disorder in type 2 diabetic db/db mice. Drug Discov. Ther..

[96] Qiao S., Liu R., Lv C., Miao Y., Yue M., Tao Y., Wei Z., Xia Y., Dai Y. (2019). Bergenin impedes the generation of extracellular matrix in glomerular mesangial cells and ameliorates diabetic nephropathy in mice by inhibiting oxidative stress via the mTOR/β-TrcP/Nrf2 pathway. Free Radic. Biol. Med..

[97] Alaofi A.L. (2020). Sinapic Acid Ameliorates the Progression of Streptozotocin (STZ)-Induced Diabetic Nephropathy in Rats via NRF2/HO-1 Mediated Pathways. Front. Pharmacol..

[98] Liu Y., Dai W., Ye S. (2019). The olive constituent oleuropein exerts nephritic protective effects on diabetic nephropathy in db/db mice. Arch. Physiol. Biochem..

[99] Hou B., Qiang G., Zhao Y., Yang X., Chen X., Yan Y., Wang X., Liu C., Zhang L., Du G. (2018). Salvianolic Acid A Protects Against Diabetic Nephropathy through Ameliorating Glomerular Endothelial Dysfunction via Inhibiting AGE-RAGE Signaling. Cell. Physiol. Biochem..

[100] Locatelli M., Zoja C., Zanchi C., Corna D., Villa S., Bolognini S., Novelli R., Perico L., Remuzzi G., Benigni A. (2020). Manipulating Sirtuin 3 pathway ameliorates renal damage in experimental diabetes. Sci. Rep..

[101] Wild C.P. (2014). International Agency for Research on Cancer. Encyclopedia of Toxicology.

[102] Hsieh J.J., Purdue M.P., Signoretti S., Swanton C., Albiges L., Schmidinger M., Heng D.Y., Larkin J., Ficarra V. (2017). Renal cell carcinoma. Nat. Rev. Dis. Prim..

[103] Mitchell T.J., Turajlic S., Rowan A., Nicol D., Farmery J.H.R., O’Brien T., Martincorena I., Tarpey P., Angelopoulos N., Yates L.R. (2018). Timing the Landmark Events in the Evolution of Clear Cell Renal Cell Cancer: TRACERx Renal. Cell.

[104] Moch H., Cubilla A.L., Humphrey P.A., Reuter V.E., Ulbright T.M. (2016). The 2016 WHO Classification of Tumours of the Urinary System and Male Genital Organs—Part A: Renal, Penile, and Testicular Tumours. Eur. Urol..

[105] Amawi H., Ashby C.R., Samuel T., Peraman R., Tiwari A.K. (2017). Polyphenolic nutrients in cancer chemoprevention and metastasis: Role of the epithelial-to-mesenchymal (EMT) pathway. Nutrients.

[106] Tian X., Zhang S., Zhang Q., Kang L., Ma C., Feng L., Li S., Li J., Yang L., Liu J. (2020). Resveratrol inhibits tumor progression by down-regulation of NLRP3 in renal cell carcinoma. J. Nutr. Biochem..

[107] Yang R., Zhang H., Zhu L. (2011). Inhibitory effect of resveratrol on the expression of the VEGF gene and proliferation in renal cancer cells. Mol. Med. Rep..

[108] Chen L., Yang S., Liao W., Xiong Y. (2015). Modification of Antitumor Immunity and Tumor Microenvironment by Resveratrol in Mouse Renal Tumor Model. Cell Biochem. Biophys..

[109] Kim C., Baek S.H., Um J.Y., Shim B.S., Ahn K.S. (2016). Resveratrol attenuates constitutive STAT3 and STAT5 activation through induction of PTPε and SHP-2 tyrosine phosphatases and potentiates sorafenib-induced apoptosis in renal cell carcinoma. BMC Nephrol..

[110] Li J., Qiu M., Chen L., Liu L., Tan G., Liu J. (2017). Resveratrol promotes regression of renal carcinoma cells via a rennin-angiotensin system suppression-dependent mechanism. Oncol. Lett..

[111] Wang K.S., Xu T.B., Ruan H.L., Xiao H.B., Liu J., Song Z.S., Cao Q., Bao L., Liu D., Wang C. (2019). LXRα promotes cell metastasis by regulating the NLRP3 inflammasome in renal cell carcinoma. Cell Death Dis..

[112] Liu Q., Fang Q., Ji S., Han Z., Cheng W., Zhang H. (2018). Resveratrol-mediated apoptosis in renal cell carcinoma via the p53/AMP-activated protein kinase/mammalian target of rapamycin autophagy signaling pathway. Mol. Med. Rep..

[113] Zhao Y., Tang H., Zeng X., Ye D., Liu J. (2018). Resveratrol inhibits proliferation, migration and invasion via Akt and ERK1/2 signaling pathways in renal cell carcinoma cells. Biomed. Pharmacother..

[114] Dai L., Chen L., Wang W., Lin P. (2020). Resveratrol inhibits ACHN cells via regulation of histone acetylation. Pharm. Biol..

[115] Yao H., Fan M., He X. (2020). Autophagy suppresses resveratrol-induced apoptosis in renal cell carcinoma 786-O cells. Oncol. Lett..

[116] Ke Y., Chen L., Zhou M., Guo J., Wang Y., Zheng D., Zhong S. (2019). Resveratrol enhances chemosensitivity of renal cell carcinoma to paclitaxel. Front. Biosci. Landmark.

[117] Kabel A.M., Atef A., Estfanous R.S. (2018). Ameliorative potential of sitagliptin and/or resveratrol on experimentally-induced clear cell renal cell carcinoma. Biomed. Pharmacother..

[118] Zeng Y., di Li F., Shi C.W., Du J.L., Xue Y.J., Liu X.Y., Cao X., Wei N. (2020). Mechanism and therapeutic prospect of resveratrol combined with TRAIL in the treatment of renal cell carcinoma. Cancer Gene Ther..

[119] Devi K.P., Rajavel T., Daglia M., Nabavi S.F., Bishayee A., Nabavi S.M. (2017). Targeting miRNAs by polyphenols: Novel therapeutic strategy for cancer. Semin. Cancer Biol..

[120] Li F., Qasim S., Li D., Dou Q.P. (2021). Updated review on green tea polyphenol epigallocatechin-3-gallate as a cancer epigenetic regulator. Semin. Cancer Biol..

[121] Gu B., Ding Q., Xia G., Fang Z. (2009). EGCG inhibits growth and induces apoptosis in renal cell carcinoma through TFPI-2 overexpression. Oncol. Rep..

[122] Chen S.J., Yao X.D., Peng B., Xu Y.F., Wang G.C., Huang J., Liu M., Zheng J.H. (2016). Epigallocatechin-3-gallate inhibits migration and invasion of human renal carcinoma cells by downregulating matrix metalloproteinase-2 and matrix metalloproteinase-9. Exp. Ther. Med..

[123] Wei R., Zhu G., Jia N., Yang W. (2015). Epigallocatechin-3-gallate Sensitizes Human 786-O Renal Cell Carcinoma Cells to TRAIL-Induced Apoptosis. Cell Biochem. Biophys..

[124] Zhang H., Xu W., Li B., Zhang K., Wu Y., Xu H., Wang J., Zhang J., Fan R., Wei J. (2015). Curcumin Promotes Cell Cycle Arrest and Inhibits Survival of Human Renal Cancer Cells by Negative Modulation of the PI3K/AKT Signaling Pathway. Cell Biochem. Biophys..

[125] Seo B.R., Min K., Cho I.J., Kim S.C., Kwon T.K. (2014). Curcumin significantly enhances dual PI3K/Akt and mTOR inhibitor NVP-BEZ235-induced apoptosis in human renal carcinoma caki cells through down-regulation of p53-dependent Bcl-2 expression and inhibition of Mcl-1 protein stability. PLoS ONE.

[126] Li G., Wang Z., Chong T., Yang J., Li H., Chen H. (2017). Curcumin enhances the radiosensitivity of renal cancer cells by suppressing NF-κB signaling pathway. Biomed. Pharmacother..

[127] Caparica R., Júlio A., Araújo M.E.M., Baby A.R., Fonte P., Costa J.G., Santos de Almeida T. (2020). Anticancer activity of rutin and its combination with ionic liquids on renal cells. Biomolecules.

[128] Hung T.W., Chen P.N., Wu H.C., Wu S.W., Tsai P.Y., Hsieh Y.S., Chang H.R. (2017). Kaempferol inhibits the invasion and migration of renal cancer cells through the downregulation of AKT and FAK pathways. Int. J. Med. Sci..

[129] Rehman M.U., Tahir M., Khan A.Q., Khan R., Lateef A., Qamar W., Ali F., Sultana S. (2013). Chrysin suppresses renal carcinogenesis via amelioration of hyperproliferation, oxidative stress and inflammation: Plausible role of NF-κB. Toxicol. Lett..

[130] Kim S.Y., Moon A. (2012). Drug-induced nephrotoxicity and its biomarkers. Biomol. Ther..

[131] McSweeney K.R., Gadanec L.K., Qaradakhi T., Ali B.A., Zulli A., Apostolopoulos V. (2021). Mechanisms of cisplatin-induced acute kidney injury: Pathological mechanisms, pharmacological interventions, and genetic mitigations. Cancers.

[132] Casanova A.G., Hernández-Sánchez M.T., Martínez-Salgado C., Morales A.I., Vicente-Vicente L., López-Hernández F.J. (2020). A meta-analysis of preclinical studies using antioxidants for the prevention of cisplatin nephrotoxicity: Implications for clinical application. Crit. Rev. Toxicol..

[133] Kim D.H., Jung Y.J., Lee J.E., Lee A.S., Kang K.P., Lee S., Park S.K., Han M.K., Lee S.Y., Ramkumar K.M. (2011). Sirt1 activation by resveratrol ameliorates cisplatin-induced renal injury through deacetylation of p53. Am. J. Physiol. Ren. Physiol..

[134] Valentovic M.A., Ball J.G., Mike Brown J., Terneus M.V., McQuade E., Van Meter S., Hedrick H.M., Roy A.A., Williams T. (2014). Resveratrol attenuates cisplatin renal cortical cytotoxicity by modifying oxidative stress. Toxicol. Vitr..

[135] Hao Q., Xiao X., Zhen J., Feng J., Song C., Jiang B., Hu Z. (2016). Resveratrol attenuates acute kidney injury by inhibiting death receptor-mediated apoptotic pathways in a cisplatin-induced rat model. Mol. Med. Rep..

[136] Do Amaral C.L., Francescato H.D.C., Coimbra T.M., Costa R.S., D’arc Castania Darin J., Antunes L.M.G., Bianchi M.D.L.P. (2008). Resveratrol attenuates cisplatin-induced nephrotoxicity in rats. Arch. Toxicol..

[137] Darwish M.A., Abo-Youssef A.M., Khalaf M.M., Abo-Saif A.A., Saleh I.G., Abdelghany T.M. (2018). Resveratrol influences platinum pharmacokinetics: A novel mechanism in protection against cisplatin-induced nephrotoxicity. Toxicol. Lett..

[138] Cigremis Y., Akgoz M., Ozen H., Karaman M., Kart A., Gecer M., Atalan G. (2015). Resveratrol ameliorates cisplatin-induced oxidative injury in New Zealand rabbits. Can. J. Physiol. Pharmacol..

[139] Kusumoto M., Kamobayashi H., Sato D., Komori M., Yoshimura M., Hamada A., Kohda Y., Tomita K., Saito H. (2011). Alleviation of cisplatin-induced acute kidney injury using phytochemical polyphenols is accompanied by reduced accumulation of indoxyl sulfate in rats. Clin. Exp. Nephrol..

[140] Kuhlmann M.K., Horsch E., Burkhardt G., Wagner M., Köhler H. (1998). Reduction of cisplatin toxicity in cultured renal tubular cells by the bioflavonoid quercetin. Arch. Toxicol..

[141] Li R., Hu L., Hu C., Wang Q., Lei Y., Zhao B. (2020). Myricitrin protects against cisplatin-induced kidney injury by eliminating excessive reactive oxygen species. Int. Urol. Nephrol..

[142] Zou P., Song J., Jiang B., Pei F., Chen B., Yang X., Liu G., Hu Z. (2014). Epigallocatechin-3-gallate protects against cisplatin nephrotoxicity by inhibiting the apoptosis in mouse. Int. J. Clin. Exp. Pathol..

[143] Chen B., Liu G., Zou P., Li X., Hao Q., Jiang B., Yang X., Hu Z. (2015). Epigallocatechin-3-gallate protects against cisplatin-induced nephrotoxicity by inhibiting endoplasmic reticulum stress-induced apoptosis. Exp. Biol. Med..

[144] El-Mowafy A.M., Salem H.A., Al-Gayyar M.M., El-Mesery M.E., El-Azab M.F. (2011). Evaluation of renal protective effects of the green-tea (EGCG) and red grape resveratrol: Role of oxidative stress and inflammatory cytokines. Nat. Prod. Res..

[145] El-Mowafy A.M., Al-Gayyar M.M., Salem H.A., El-Mesery M.E., Darweish M.M. (2010). Novel chemotherapeutic and renal protective effects for the green tea (EGCG): Role of oxidative stress and inflammatory-cytokine signaling. Phytomedicine.

[146] Malik S., Suchal K., Bhatia J., Gamad N., Dinda A.K., Gupta Y.K., Arya D.S. (2016). Molecular mechanisms underlying attenuation of cisplatin-induced acute kidney injury by epicatechin gallate. Lab. Investig..

[147] Waseem M., Kaushik P., Parvez S. (2013). Mitochondria-mediated mitigatory role of curcumin in cisplatin-induced nephrotoxicity. Cell Biochem. Funct..

[148] Kumar P., Sulakhiya K., Barua C.C., Mundhe N. (2017). TNF-α, IL-6 and IL-10 expressions, responsible for disparity in action of curcumin against cisplatin-induced nephrotoxicity in rats. Mol. Cell. Biochem..

[149] Sahin K., Orhan C., Tuzcu M., Muqbil I., Sahin N., Gencoglu H., Guler O., Padhye S.B., Sarkar F.H., Mohammad R.M. (2014). Comparative in vivo evaluations of curcumin and its analog difluorinated curcumin against cisplatin-induced nephrotoxicity. Biol. Trace Elem. Res..

[150] Trujillo J., Molina-Jijón E., Medina-Campos O.N., Rodríguez-Muñoz R., Reyes J.L., Loredo M.L., Barrera-Oviedo D., Pinzón E., Rodríguez-Rangel D.S., Pedraza-Chaverri J. (2016). Curcumin prevents cisplatin-induced decrease in the tight and adherens junctions: Relation to oxidative stress. Food Funct..

[151] Ortega-Domínguez B., Aparicio-Trejo O.E., García-Arroyo F.E., León-Contreras J.C., Tapia E., Molina-Jijón E., Hernández-Pando R., Sánchez-Lozada L.G., Barrera-Oviedo D., Pedraza-Chaverri J. (2017). Curcumin prevents cisplatin-induced renal alterations in mitochondrial bioenergetics and dynamic. Food Chem. Toxicol..

[152] Ueki M., Ueno M., Morishita J., Maekawa N. (2013). Curcumin ameliorates cisplatin-induced nephrotoxicity by inhibiting renal inflammation in mice. J. Biosci. Bioeng..

[153] Ugur S., Ulu R., Dogukan A., Gurel A., Yigit I.P., Gozel N., Aygen B., Ilhan N. (2015). The renoprotective effect of curcumin in cisplatin-induced nephrotoxicity. Ren. Fail..

[154] Al-Kharusi N., Babiker H.A., Al-Salam S., Waly M.I., Nemmar A., Al-Lawati I., Yasin J., Beegam S., Ali B.H. (2013). Ellagic acid protects against cisplatin-induced nephrotoxicity in rats: A dose-dependent study. Eur. Rev. Med. Pharmacol. Sci..

[155] Goyal Y., Koul A., Ranawat P. (2019). Ellagic acid ameliorates cisplatin toxicity in chemically induced colon carcinogenesis. Mol. Cell. Biochem..

[156] Ahmad S.T., Sultana S. (2012). Tannic acid mitigates cisplatin-induced nephrotoxicity in mice. Hum. Exp. Toxicol..

[157] Chien L.H., Wu C.T., Deng J.S., Jiang W.P., Huang W.C., Huang G.J. (2021). Salvianolic acid c protects against cisplatin-induced acute kidney injury through attenuation of inflammation, oxidative stress and apoptotic effects and activation of the CaMKK–AMPK–sirt1-associated signaling pathway in mouse models. Antioxidants.

[158] Wang T.E.J., Liu H.T., Lai Y.H., Jan T.R., Nomura N., Chang H.W., Chou C.C., Lee Y.J., Tsai P.S.J. (2018). Honokiol, a polyphenol natural compound, attenuates cisplatin-induced acute cytotoxicity in renal epithelial cells through cellular oxidative stress and cytoskeleton modulations. Front. Pharmacol..

[159] Liu H.T., Wang T.E., Hsu Y.T., Chou C.C., Huang K.H., Hsu C.C., Liang H.J., Chang H.W., Lee T.H., Tsai P.S. (2019). Nanoparticulated honokiol mitigates cisplatin-induced chronic kidney injury by maintaining mitochondria antioxidant capacity and reducing caspase 3-associated cellular apoptosis. Antioxidants.

[160] Aladaileh S.H., Al-Swailmi F.K., Abukhalil M.H., Ahmeda A.F., Mahmoud A.M. (2021). Punicalagin prevents cisplatin-induced nephrotoxicity by attenuating oxidative stress, inflammatory response, and apoptosis in rats. Life Sci..

[161] Chen C., Ai Q., Wei Y. (2021). Hydroxytyrosol protects against cisplatin-induced nephrotoxicity via attenuating CKLF1 mediated inflammation, and inhibiting oxidative stress and apoptosis. Int. Immunopharmacol..

[162] Vicente-Vicente L., Casanova A.G., Hernández-Sánchez M.T., Pescador M., López-Hernández F.J., Morales A.I. (2017). A systematic meta-analysis on the efficacy of pre-clinically tested nephroprotectants at preventing aminoglycoside nephrotoxicity. Toxicology.

[163] Morales A.I., Buitrago J.M., Santiago J.M., Fernández-Tagarro M., López-Novoa J.M., Pérez-Barriocanal F. (2002). Protective effect of trans-Resveratrol on gentamicin-induced nephrotoxicity. Antioxid. Redox Signal..

[164] Silan C., Uzun Ö., Çomunoǧlu N.Ü., Gokçen S., Bedirhan S., Cengiz M. (2007). Gentamicin-induced nephrotoxicity in rats ameliorated and healing effects of resveratrol. Biol. Pharm. Bull..

[165] Beshay O.N., Ewees M.G., Abdel-Bakky M.S., Hafez S.M.N.A., Abdelrehim A.B., Bayoumi A.M.A. (2020). Resveratrol reduces gentamicin-induced EMT in the kidney via inhibition of reactive oxygen species and involving TGF-β/Smad pathway. Life Sci..

[166] Morales A.I., Rodríguez-Barbero A., Vicente-Sánchez C., Mayoral P., López-Novoa J.M., Pérez-Barriocanal F. (2006). Resveratrol inhibits gentamicin-induced mesangial cell contraction. Life Sci..

[167] Negrette-Guzmán M., García-Niño W.R., Tapia E., Zazueta C., Huerta-Yepez S., León-Contreras J.C., Hernández-Pando R., Aparicio-Trejo O.E., Madero M., Pedraza-Chaverri J. (2015). Curcumin Attenuates Gentamicin-Induced Kidney Mitochondrial Alterations: Possible Role of a Mitochondrial Biogenesis Mechanism. Evid. Based Complement. Altern. Med..

[168] Ansari M.A., Raish M., Ahmad A., Ahmad S.F., Mudassar S., Mohsin K., Shakeel F., Korashy H.M., Bakheet S.A. (2016). Sinapic acid mitigates gentamicin-induced nephrotoxicity and associated oxidative/nitrosative stress, apoptosis, and inflammation in rats. Life Sci..

[169] Dallak M., Dawood A.F., Haidara M.A., Abdel Kader D.H., Eid R.A., Kamar S.S., Shams Eldeen A.M., Al-Ani B. (2020). Suppression of glomerular damage and apoptosis and biomarkers of acute kidney injury induced by acetaminophen toxicity using a combination of resveratrol and quercetin. Drug Chem. Toxicol..

[170] Liu T., Liu S., Yu X., Song N., Xu X., Hu J., Zhang T., Ding X. (2016). Salvianolic acid B prevents iodinated contrast media-induced acute renal injury in rats via the PI3K/Akt/Nrf2 pathway. Oxid. Med. Cell. Longev..

[171] Vesely O., Baldovska S., Kolesarova A. (2021). Enhancing Bioavailability of Nutraceutically Used Resveratrol and Other Stilbenoids. Nutrients.

[172] Walle T., Hsieh F., DeLegge M.H., Oatis J.E., Walle U.K. (2004). High absorption but very low bioavailability of oral resveratrol in humans. Drug Metab. Dispos..

[173] Mcdade D., Patrick R.M., Rizvi S.S.H., Knorr D., Labuza T.P., Tomás-Barberán F.A., González-Sarrías A., García-Villalba R. (2021). Dietary Polyphenols: Metabolism and Health Effects.

[174] Di Lorenzo C., Colombo F., Biella S., Stockley C., Restani P. (2021). Polyphenols and human health: The role of bioavailability. Nutrients.

[175] Manach C., Scalbert A., Morand C., Rémésy C., Jiménez L. (2004). Polyphenols: Food sources and bioavailability. Am. J. Clin. Nutr..

[176] D’Archivio M., Filesi C., Varì R., Scazzocchio B., Masella R. (2010). Bioavailability of the polyphenols: Status and controversies. Int. J. Mol. Sci..

[177] Marín L., Miguélez E.M., Villar C.J., Lombó F. (2015). Bioavailability of dietary polyphenols and gut microbiota metabolism: Antimicrobial properties. BioMed Res. Int..

[178] Teng H., Chen L. (2019). Polyphenols and bioavailability: An update. Crit. Rev. Food Sci. Nutr..

[179] Carregosa D., Mota S., Ferreira S., Alves-Dias B., Loncarevic-Vasiljkovic N., Crespo C.L., Menezes R., Teodoro R., Santos C.N. (2021). Dos Overview of beneficial effects of (Poly)phenol metabolites in the context of neurodegenerative diseases on model organisms. Nutrients.

[180] Kay C.D., Pereira-Caro G., Ludwig I.A., Clifford M.N., Crozier A. (2017). Anthocyanins and Flavanones Are More Bioavailable than Previously Perceived: A Review of Recent Evidence. Annu. Rev. Food Sci. Technol..

[181] Marhuenda-Muñoz M., Laveriano-Santos E.P., Tresserra-Rimbau A., Lamuela-Raventós R.M., Miriam M.-H., Vallverdú-Quera A. (2019). Microbial Phenolic Metabolites: Which Molecules Actually Have an Effect on Human Health?. Nutrients.

[182] Selma M.V., Espín J.C., Tomás-Barberán F.A. (2009). Interaction between phenolics and gut microbiota: Role in human health. J. Agric. Food Chem..

[183] Ito H., Gonthier M.-P., Manach C., Morand C., Mennen L., Rémésy C., Scalbert A. (2005). Polyphenol levels in human urine after intake of six different polyphenol-rich beverages. Br. J. Nutr..

[184] Gomes A., Godinho-Pereira J., Oudot C., Sequeira C.O., Macià A., Carvalho F., Motilva M.J., Pereira S.A., Matzapetakis M., Brenner C. (2020). Berry fruits modulate kidney dysfunction and urine metabolome in Dahl salt-sensitive rats. Free Radic. Biol. Med..

[185] Luca S.V., Macovei I., Bujor A., Miron A., Skalicka-Woźniak K., Aprotosoaie A.C., Trifan A. (2020). Bioactivity of dietary polyphenols: The role of metabolites. Crit. Rev. Food Sci. Nutr..

[186] Masereeuw R., Russel F.G.M. (2001). Mechanisms and clinical implications of renal drug excretion. Drug Metab. Rev..

[187] Rothwell J.A., Perez-Jimenez J., Neveu V., Medina-Remón A., M’Hiri N., García-Lobato P., Manach C., Knox C., Eisner R., Wishart D.S. (2013). Phenol-Explorer 3.0: A major update of the Phenol-Explorer database to incorporate data on the effects of food processing on polyphenol content. Database.

[188] Bento A., Giacomoni F., Pavot B., Fillatre Y., Rothwell J., Sualdea B.B., Veyrat C., Gladine C., Kopec R., Bento A. PhytoHub V1.4: A New Release for the Online Database Dedicated to Food Phytochemicals and Their Human Metabolites. Proceedings of the International Conference on Food Bioactives & Health.

[189] Jain R.B. (2017). Trends in the levels of urine and serum creatinine: Data from NHANES 2001–2014. Environ. Sci. Pollut. Res..

[190] Schoen T., Blum J., Paccaud F., Burnier M., Bochud M., Conen D. (2013). Factors associated with 24-hour urinary volume: The Swiss salt survey. BMC Nephrol..

[191] Roowi S., Stalmach A., Mullen W., Lean M.E.J., Edwards C.A., Crozier A. (2010). Green tea flavan-3-ols: Colonic degradation and urinary excretion of catabolites by humans. J. Agric. Food Chem..

[192] Urpi-Sarda M., Llorach R., Khan N., Monagas M., Rotches-Ribalta M., Lamuela-Raventos R., Estruch R., Tinahones F.J., Andres-Lacueva C. (2010). Effect of milk on the urinary excretion of microbial phenolic acids after cocoa powder consumption in humans. J. Agric. Food Chem..

[193] Olthof M.R., Hollman P.C.H., Buijsman M.N.C.P., Van Amelsvoort J.M.M., Katan M.B. (2003). Chlorogenic acid, quercetin-3-rutinoside and black tea phenols are extensively metabolized in humans. J. Nutr..

[194] Loke W.M., Jenner A.M., Proudfoot J.M., McKinley A.J., Hodgson J.M., Halliwell B., Croft K.D. (2009). A metabolite profiling approach to identify biomarkers of flavonoid intake in humans. J. Nutr..

[195] Baba S., Osakabe N., Natsume M., Yasuda A., Muto Y., Hiyoshi K., Takano H., Yoshikawa T., Terao J. (2005). Absorption, metabolism, degradation and urinary excretion of rosmarinic acid after intake of Perilla frutescens extract in humans. Eur. J. Nutr..

[196] Feliciano R.P., Istas G., Heiss C., Rodriguez-Mateos A. (2016). Plasma and urinary phenolic profiles after acute and repetitive intake of wild blueberry. Molecules.

[197] Urpi-Sarda M., Monagas M., Khan N., Llorach R., Lamuela-Raventós R.M., Jáuregui O., Estruch R., Izquierdo-Pulido M., Andrés-Lacueva C. (2009). Targeted metabolic profiling of phenolics in urine and plasma after regular consumption of cocoa by liquid chromatography-tandem mass spectrometry. J. Chromatogr. A.

[198] Visioli F., Galli C., Bornet F., Mattei A., Patelli R., Galli G., Caruso D. (2000). Olive oil phenolics are dose-dependently absorbed in humans. FEBS Lett..

[199] Castello F., Costabile G., Bresciani L., Tassotti M., Naviglio D., Luongo D., Ciciola P., Vitale M., Vetrani C., Galaverna G. (2018). Bioavailability and pharmacokinetic profile of grape pomace phenolic compounds in humans. Arch. Biochem. Biophys..

[200] Visioli F., Caruso D., Galli C., Viappiani S., Galli G., Sala A. (2000). Olive oils rich in natural catecholic phenols decrease isoprostane excretion in humans. Biochem. Biophys. Res. Commun..

[201] Feliciano R.P., Boeres A., Massacessi L., Istas G., Ventura M.R., Nunes Dos Santos C., Heiss C., Rodriguez-Mateos A. (2016). Identification and quantification of novel cranberry-derived plasma and urinary (poly)phenols. Arch. Biochem. Biophys..

[202] De Ferrars R.M., Czank C., Zhang Q., Botting N.P., Kroon P.A., Cassidy A., Kay C.D. (2014). The pharmacokinetics of anthocyanins and their metabolites in humans. Br. J. Pharmacol..

[203] Lea-Henry T.N., Carland J.E., Stocker S.L., Sevastos J., Roberts D.M. (2018). Clinical pharmacokinetics in kidney disease: Fundamental principles. Clin. J. Am. Soc. Nephrol..

[204] Mahmoodpoor F., Rahbar Saadat Y., Barzegari A., Ardalan M., Zununi Vahed S.V. (2017). The impact of gut microbiota on kidney function and pathogenesis. Biomed. Pharmacother..

